# SOCS3 deficiency in cardiomyocytes elevates sensitivity of ischemic preconditioning that synergistically ameliorates myocardial ischemia reperfusion injury

**DOI:** 10.1371/journal.pone.0254712

**Published:** 2021-07-22

**Authors:** Shoichiro Nohara, Mai Yamamoto, Hideo Yasukawa, Takanobu Nagata, Jinya Takahashi, Koutatsu Shimozono, Toshiyuki Yanai, Tomoko Sasaki, Kota Okabe, Tatsuhiro Shibata, Daiki Akagaki, Kazutoshi Mawatari, Yoshihiro Fukumoto

**Affiliations:** 1 Division of Cardiovascular Medicine, Department of Internal Medicine, Kurume University School of Medicine, Kurume, Japan; 2 Cardiovascular Research Institute, Kurume University, Kurume, Japan; Indiana University School of Medicine, UNITED STATES

## Abstract

Ischemic preconditioning (IPC) is the most powerful endogenous cardioprotective form of cellular adaptation. However, the inhibitory or augmenting mechanism underlying cardioprotection via IPC remains largely unknown. Suppressor of cytokine signaling-3 (SOCS3) is a cytokine-inducible potent negative feedback regulator of the signal transducer and activator of transcription-3 (STAT3) signaling pathway. Here, we aimed to determine whether cardiac SOCS3 deficiency and IPC would synergistically reduce infarct size after myocardial ischemia reperfusion injury. We evaluated STAT3 activation and SOCS3 induction after ischemic conditioning (IC) using western blot analysis and real-time PCR, and found that myocardial IC alone transiently activated myocardial STAT3 and correspondingly induced SOCS3 expression in wild-type mice. Compared with wild-type mice, cardiac-specific SOCS3 knockout (SOCS3-CKO) mice showed significantly greater and more sustained IC-induced STAT3 activation. Following ischemia reperfusion, IPC substantially reduced myocardial infarct size and significantly enhanced STAT3 phosphorylation in SOCS3-CKO mice compared to in wild-type mice. Real-time PCR array analysis revealed that SOCS3-CKO mice after IC exhibited significantly increased expressions of several anti-apoptotic genes and SAFE pathway-related genes. Moreover, real-time PCR analysis revealed that myocardial IC alone rapidly induced expression of the STAT3-activating cytokine erythropoietin in the kidney at 1 h post-IC. We also found that the circulating erythropoietin level was promptly increased at 1 h after myocardial IC. Myocardial SOCS3 deficiency and IPC exert synergistic effects in the prevention of myocardial injury after ischemia reperfusion. Our present results suggest that myocardial SOCS3 is a potent inhibitor of IPC-induced cardioprotection, and that myocardial SOCS3 inhibition augment IPC-mediated cardioprotection during ischemia reperfusion injury.

## Introduction

Among patients with acute myocardial infarction (MI), prognosis has been improved by the development of reperfusion therapy via percutaneous coronary intervention or thrombolysis; however, the incidence of post-MI heart failure has been increasing [1–6]. After acute MI, the infarct size determines the development of left ventricular remodeling and heart failure. Timely reperfusion therapy is the most effective treatment of minimizing the infarct size and improving clinical outcome [1–6]. However, paradoxically, reperfusion itself, induces further myocardial injury—generally known as myocardial ischemia reperfusion (I/R) injury. Thus, there is a need for new strategies to reduce infarct size during I/R in MI patients.

Murry et al [7] first demonstrated that myocardial infarct size could be reduced by brief episodes of nonlethal ischemia and reperfusion before sustained ischemia. This conditioning phenomenon has been termed ischemic preconditioning (IPC), and is the most powerful endogenous cardioprotective form of cellular adaptation that has been reproduced in numerous species and demonstrated in noncardiac tissue, including liver, kidney, lung, and intestine [1, 3, 8–10]. However, the inhibitory or augmenting mechanism underlying IPC-induced cardioprotection remains largely unknown.

The *janus* kinase (JAK)–signal transducer and activator of transcription-3 (STAT3) pathway is a potent pro-survival signaling pathway during stress-induced myocardial injury, including acute MI [11–17]. The survival activating factor enhancement (SAFE) pathway and the reperfusion injury salvage kinase (RISK) pathway have been identified as major signal transduction pathways that are causally involved in IPC’s cardioprotection. STAT3 is a central element of SAFE pathway and AKT and ERK1/2 are central elements of RISK pathway [6, 8, 18]. Evidence from animal studies suggests that STAT3 signaling pathway activation is crucial for achieving cardioprotection through IPC [12, 18–23]. Smith et al [22] reported that genetic depletion of STAT3 in cardiomyocytes abolishes the capacity to activate IPC during I/R. Heusch and colleagues [24] showed that myocardial STAT3 plays a causal role in IPC-based cardioprotection in large animals, suggesting that the STAT3 signaling pathway is essential for IPC-mediated cardioprotection during I/R.

Our group and others have identified suppressor of cytokine signaling (SOCS) family proteins as cytokine-inducible highly specific and potent inhibitors of JAK–STAT signaling pathways [25–27]. We previously demonstrated that SOCS3 acts as a pseudosubstrate, strongly inhibiting JAK–STAT-mediated cytokine signaling pathways by interacting with JAK and inhibiting JAK activity [28, 29]. SOCS3 expression is induced by JAK–STAT-activating cytokines including erythropoietin (EPO) and granulocyte colony-stimulating factor (G-CSF), and by myocardial insults, such as viral infection, pressure overload, or ischemia [30–34]. We previously reported that the forced SOCS3 expression inhibits cytokine-promoted cardiomyocyte survival in vitro [33], and that transgenic cardiac-specific SOCS3 expression facilitates coxsackievirus-induced cardiac injury in mice [32]. In contrast, myocardial SOCS3 deletion in mice enhances the activation of multiple cardioprotective signaling pathways and prevents post-MI heart failure and myocardial I/R injury [30, 31]. During I/R injury, cardiac-specific SOCS3 knockout (SOCS3-CKO) mice exhibit sustained phosphorylation of STAT3, AKT, and ERK1/2; prevention of myocardial apoptosis and injury; and augmented expression of the anti-apoptotic myeloid cell leukemia-1 (Mcl-1) [30]. Therefore, we hypothesized that the myocardial SOCS3 inhibition would augment IPC-mediated cardioprotection during I/R. To test this hypothesis, we applied cardiac IPC in the myocardial I/R injury model using SOCS3-CKO mice, and investigated the role of myocardial SOCS3 in the IPC-induced cardioprotection during I/R injury.

## Methods

### Ethics statement

All experimental protocols involving animal were approved by the Animal Experiments Review Boards of Kurume University (Permit Number: 2017–075, 2018–156, 2019–107, 2020-067-1). All procedures on the mice were performed under general anesthesia with isoflurane (5% in 100% oxygen for induction; 1–2% in 100% oxygen for maintenance) using an animal anesthesia machine (model TK-5, Bio Machinery, Chiba, Japan), and all efforts were made to minimize suffering. At the end of experimental periods, mice were euthanized through deep isoflurane anesthesia.

### Cardiac-specific SOCS3 knockout mice

The mice were maintained with normal chow and freely available drinking water. This study utilized 8- to 12-week-old male mice in a Balb/c background. To determine the tissue-specific roles of SOCS3, we generated SOCS3-flox mice as previously described [35]. To investigate the role of JAK–STAT signaling and its negative regulator SOCS3 in myocardial IPC, SOCS3-flox mice were bred with mice harboring a transgene encoding Cre recombinase driven by the α-myosin heavy chain (αMHC)-promoter [31, 36]. We confirmed the marked reduction of SOCS3 mRNA and protein in SOCS3-CKO hearts during myocardial I/R injury, as previously described [30]. SOCS3-flox littermate mice lacking the αMHC-Cre transgene served as wild-type (WT) control animal.

### In vivo mouse model of myocardial ischemia reperfusion injury

We used an open-chest in situ model to study I/R injury, as previously described [30, 37]. Briefly, mice were anesthetized using inhaled isoflurane administered via an endotracheal tube, and were provided positive-pressure ventilation using a constant-volume ventilator operating on the Starling principle (HSE MiniVent, Harvard Apparatus GmbH). After opening the thoracic cavity by left thoracotomy, and then an 8–0 prolene suture was passed under the left anterior descending (LAD) coronary artery at the inferior edge of the left atrium, and then tied to produce an occlusion. Ischemia was confirmed based on blanching downstream of the ligation, and persistent ST segment elevation on the electrocardiogram. Body temperature was maintained at 37°C using a heating pad, and temperature was monitored using a rectal thermometer. After 60 min of ischemia, the ligature was released. Reperfusion of the LAD coronary artery reperfusion was confirmed based on color restoration in the ischemic myocardium, and T-wave inversion on electrocardiogram. The chest was closed using continuous 6–0 prolene sutures, and the endotracheal tube was removed following resumption of respiration.

### IC or IPC induction

As was done for the in vivo myocardial I/R injury protocol, we anesthetized mice using inhaled isoflurane, provided positive-pressure ventilation, opened the thoracic cavity, and visualized the LAD coronary artery. Next, IC or IPC was induced by three cycles comprising 5 min of coronary artery occlusion followed by 5 min of reperfusion. IC and IPC were performed following the exact same experimental procedure. Throughout the manuscript, we use the term IC when describing experiments without 1-hour ischemia, and IPC when describing experiments including 1-hour ischemia.

### Evans blue dye and triphenyltetrazolium chloride staining

At 24 hours post-reperfusion, each mouse was anesthetized as described above. Then chest was re-opened, and the LAD coronary artery re-occluded. The heart was perfused with 5% Evans blue dye, which stained the normally perfused area, such that an absence of staining indicated the ischemic area—i.e., the area at risk (AAR). Next the heart was excised, and the left ventricle (LV) was cut into five transverse slices from apex to base. These slices were incubated in 1% triphenyltetrazolium chloride (TTC) solution at 37°C for 10 min, photographed with a digital camera (Leica, M165; Wetzlar, Germany), and weighed. On each image, we measured the infarct area (i.e., the area lacking TTC staining) and the AAR and LV areas, using a planimeter with Image-Pro PLUS software (version 7.0J). For each slice, we determined the ratios of infarct to LV area and AAR to LV area, and multiplied these ratios by the slice weight to calculate net infarct area and AAR weights, respectively. We then summed these values for all slices. The total infarct area weight was divided by the total AAR weight (infarct area/AAR) to obtain infarct size, and total AAR weight was divided by the total LV weight (AAR/LV) to obtain ischemic size [30, 37]. We compared the infarct size in the LV at 24 hours post reperfusion among WT mice without IPC (n = 13), WT mice with IPC (n = 8), SOCS3-CKO mice without IPC (n = 7) and SOCS3-CKO mice with IPC (n = 8).

### Western blot analysis

At specific time-points after IC or after I/R, tissues were collected and homogenized in lysis buffer containing 25 mM Hepes (pH 7.5), 1% Triton X100, 150 mM NaCl, 10% glycerol, 1 mM sodium orthovanadate, 50 mM NaF, 10 mM sodium pyrophosphate, and protease inhibitor cocktail (Sigma Aldrich, St Louis, MO). Equal amounts of proteins were separated by denaturing SDS-PAGE, and then transferred onto nitrocellulose membrane (Thermo Fisher Scientific, Waltham, MA). These membranes were probed using primary antibody, and then incubated with HRP-conjugated secondary antibody. Protein signals were detected using the ECL plus system (GE Healthcare, Chicago, IL). Expression levels were determined from band intensities using Bio-ID software (Vilber Lourmat, Collégien, France), and values were expressed relative to GAPDH signals [30, 31, 37]. From Cell Signaling Technology (Beverly, MA), we purchased antibodies against phosphorylated STAT3 (P-STAT3; #9145, D3A7, rabbit monoclonal, 1:200 dilution), phosphorylated AKT (P-AKT; #4060, D9E, rabbit monoclonal, 1:200 dilution), and phosphorylated ERK1/2 (P-ERK1/2; #4370, D13.14.4E, rabbit monoclonal, 1:200 dilution). The antibody against glyceraldehyde 3-phosphate dehydrogenase (GAPDH; MAB374, 6C5, mouse monoclonal, 1:5000 dilution) was purchased from Merck Millipore (Darmstadt, Germany).

### Real-time PCR

Total RNA was isolated from heart and kidney tissues using TRIzol (Thermo Fisher Scientific, Waltham, MA), as previously described [30, 31, 37], and 1 μg of total RNA was converted into cDNA. Real-time polymerase chain reaction (PCR) assays were performed to assess the gene expressions of mouse SOCS3, EPO and GAPDH, using the corresponding primer pairs (#Mm00545913_s1, #Mm01202755_m1 and #Mm99999915_g1, respectively; Thermo Fisher Scientific, Waltham, MA,) and the StepOnePlus Real-Time PCR System (Thermo Fisher Scientific, Waltham, MA). We obtained apoptosis expression profiles using the RT2 Profiler PCR array for murine apoptosis (Qiagen, Hilden, Germany), following the manufacturer’s instructions. PCR was performed using the StepOne real-time PCR system (Thermo Fisher Scientific, Waltham, MA), and the ΔΔCt method was applied to analyze the expression levels of each gene. We evaluated the dissociation curve for each gene, and excluded genes with nonspecific amplification or undetectable expression. The gene expression profiles were displayed as a heat map created using the QIAGEN web portal at Gene Globe [31, 37].

### ELISA for EPO

Mouse serum samples were analyzed for EPO using an enzyme-linked immunosorbent assay (ELISA; R&D Systems, Minneapolis, MN), following the manufacturer’s instructions [38]. We compared the serum level of EPO before and after IC (n = 6–7 per group).

### Histological analysis

Freshly isolated hearts were fixed in 4% paraformaldehyde (PFA), dehydrated, embedded in paraffin, and sectioned. Five-micrometer sections were stained with hematoxylin and eosin or Mallory-AZAN staining.

### Echocardiogram

Mice were placed under light anesthesia with isoflurane and subjected to echocardiography as previously described [30, 31]. Transthoracic echocardiography was performed using a Vevo770 ultrasound machine (VisualSonics Inc, Toronto, Canada) equipped with a 30-MHz probe. Recording was performed as previously described [30, 31].

### Statistics

Data are expressed as the mean ± standard error of the mean (SE). Statistical analyses were performed using JMPpro12. We performed multiple group comparisons using the Kruskal–Wallis test, followed by the Dunn’s test, and comparisons between two groups were performed using the Wilcoxon rank-sum test. A *P* value of less than 0.05 was considered to indicate statistical significance.

## Results

### Ischemic conditioning alone activates myocardial STAT3 and induces SOCS3 expression

**Fig 1:**
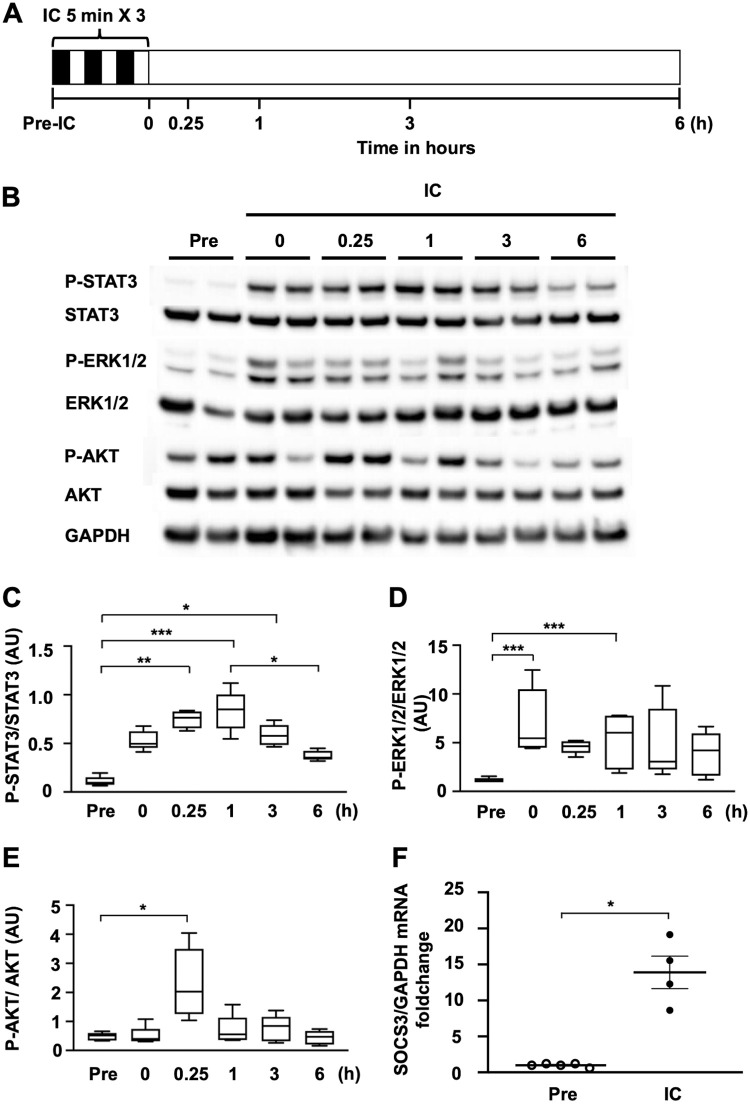
Transient myocardial STAT3 activation and SOCS3 expression in WT mice after ischemic conditioning alone. Western blot analysis of total cell lysates prepared from heart tissue of WT or SOCS3-CKO mice at the indicated time-points after IC. (**A**) Schematic illustration depicting the experimental protocol. (**B)** Blots were probed using antibodies against phosphorylated STAT3 (P-STAT3), total STAT3, phosphorylated ERK1/2 (P-ERK1/2), total ERK1/2, phosphorylated AKT (P-AKT), total AKT, and GAPDH. (**C)**, (**D)**, and (**E)** Graphs display the quantitative differences in expression between the ratios of P-STAT3 to total STAT3 (**C**), P-ERK1/2 to total ERK1/2 (**D**), and P-AKT to total AKT (**E**) (n = 5 per group). *p < 0.05, ***p < 0.01 (Dunn’s test). **(F)** Real-time PCR analysis of SOCS3 mRNA expression in mouse hearts pre-IC or 3 h after IC (n = 4–5 for each group). *p <0.05 (Wilcoxon rank-sum test). WT, wild-type; IC, ischemic conditioning; AU, arbitrary units; GAPDH, glyceraldehyde 3-phosphate dehydrogenase.

Before and during IC alone, we performed western blot analysis to assess the phosphorylation of cardioprotective signaling molecules downstream of JAK in WT mice (Fig 1A and 1B). Myocardial STAT3 phosphorylation was undetectable before IC, increased at 0 h, peaked 1 h post-IC, and then significantly decreased at 6 h post-IC (Fig 1C). Myocardial ERK1/2 phosphorylation was faint before IC and peaked at 0 h post-IC (Fig 1D). Myocardial AKT phosphorylation was faint before IC and increased at 15 m after IC (Fig 1E). IC rapidly activated the STAT3 signaling pathway, followed by significant decrease of these activation (Fig 1B and 1C), suggesting a mechanism that inhibits STAT3 activation during IC. Real-time PCR revealed that, at 3 h post-IC, myocardial SOCS3 mRNA expression was markedly increased in WT mice (Fig 1F).

### Sustained activation of STAT3 in SOCS3-CKO mice during ischemic conditioning

**Fig 2:**
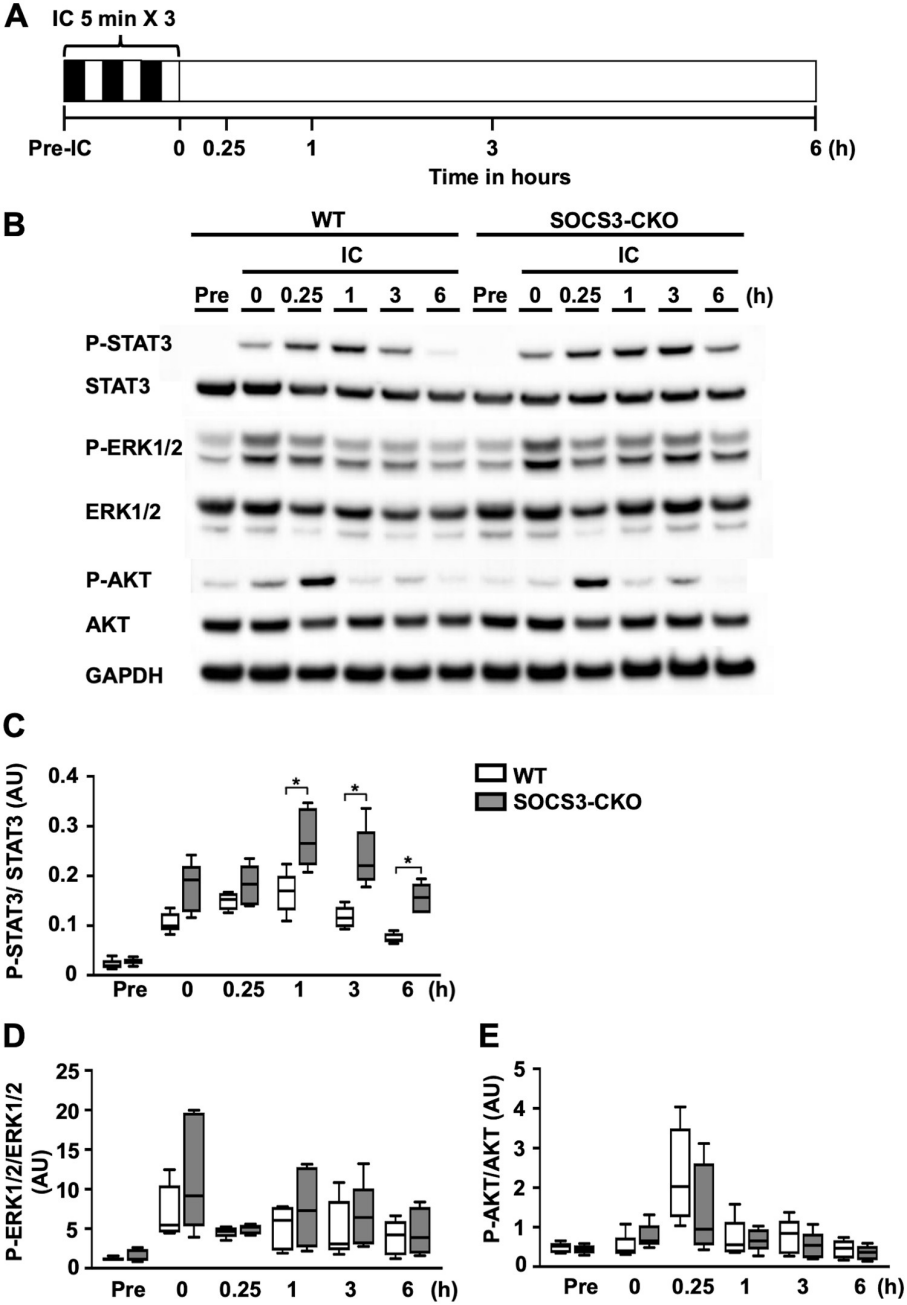
Sustained activation of STAT3 in SOCS3-CKO mice after ischemic conditioning alone. Western blot analysis of total cell lysates prepared from heart tissue of WT or SOCS3-CKO mice at the indicated time-points after IC. (**A**) Schematic illustration depicting the experimental protocol. (**B)** Blots were probed using antibodies against phosphorylated STAT3 (P-STAT3), total STAT3, phosphorylated AKT (P-AKT), total AKT, phosphorylated ERK1/2 (P-ERK1/2), total ERK1/2, and GAPDH. (**C)**, (**D)**, and (**E)** Graphs display the quantitative differences in the ratios of the expression of P-STAT3 to total STAT3 (**C**), P-ERK1/2 to total ERK1/2 (**D**), and P-AKT to total AKT (**E**) (n = 5 per group). *p < 0.05, **p < 0.01 (Wilcoxon rank-sum test). WT, wild-type; IC, ischemic conditioning; AU, arbitrary units; GAPDH, glyceraldehyde 3-phosphate dehydrogenase.

We next performed western blot analysis to compare the activation of cardioprotective signaling pathways—including STAT3, ERK1/2, and AKT—during IC in WT mice and SOCS3-CKO mice (Fig 2A and 2B). At 0 h and 15min after IC, myocardial STAT3 phosphorylation levels were similar between WT and SOCS3-CKO mice. In WT mice, STAT3 phosphorylation decreased from 3 to 6 h post-IC. In contrast, in SOCS3-CKO mice, STAT3 phosphorylation from 1 to 6 h post-IC was significantly higher compared to in WT mice (Fig 2C). ERK1/2 and AKT phosphorylation patterns were comparable between in WT and SOCS3-CKO mice (Fig 2D and 2E).

### Substantial reduction of myocardial infarct size via ischemic preconditioning during ischemia reperfusion in SOCS3-CKO mice

**Fig 3:**
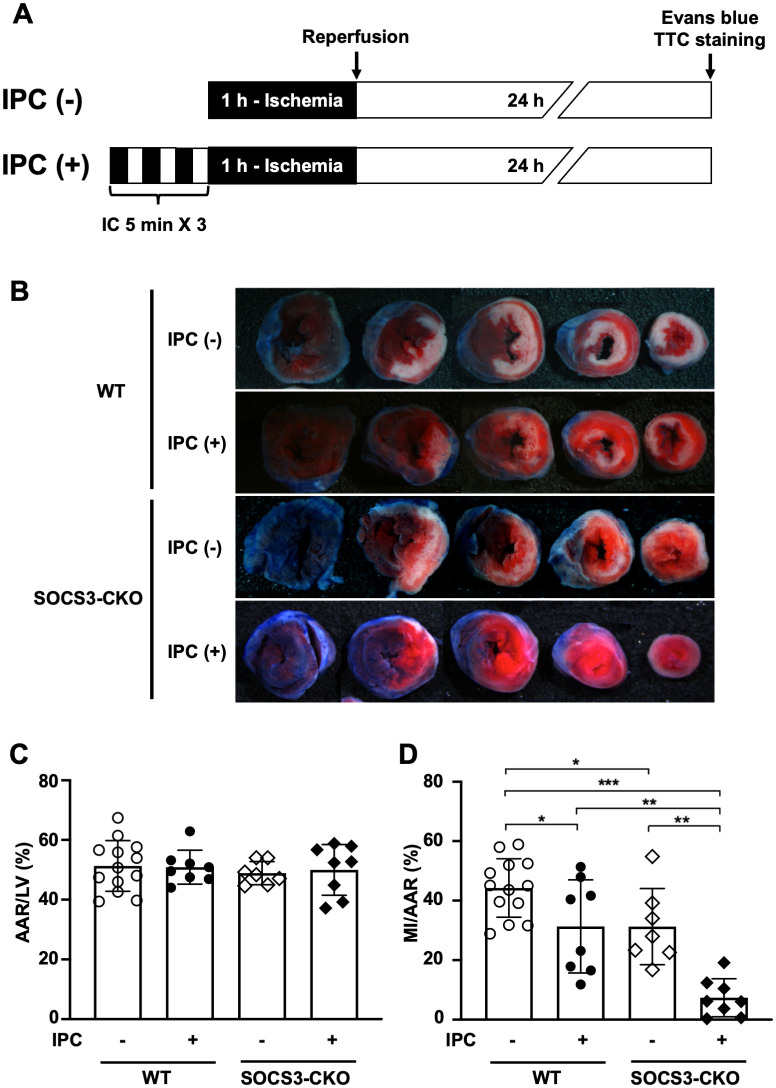
Substantial reduction of infarct size via ischemic preconditioning after I/R in SOCS3-CKO mice. (**A)** Schematic illustration depicting the experimental protocol. IPC was performed with three cycles of 5-min ischemia and 5-min reperfusion. The LAD coronary artery was occluded for 1 h, and then reperfused for 24 h. (**B)** Representative images of Evans blue and TTC staining in WT and SOCS3-CKO mice, with or without IPC, at 24 h after reperfusion (n = 7–13 per group). (**C)** and **(D)** The infarct size of the LV was expressed as a percentage of the AAR of each group. Graphs show quantification of AAR/LV (**C**) and infarct area/AAR (**D**). *P<0.05, **P<0.01, ***P<0.001 (Dunn’s test). I/R, ischemia reperfusion; IPC, ischemic preconditioning; LAD, left anterior descending; TTC, triphenyltetrazolium chloride; WT, wild-type; LV, left ventricle; AAR, area at risk.

Next we investigated how myocardial SOCS3 deficiency affected IPC-induced cardioprotection during myocardial I/R injury. Myocardial IPC was elicited by three cycles of 5-min LAD coronary artery occlusion and 5-min reperfusion, and myocardial injury was induced by 1-h ligation of the LAD coronary artery. At 24 h post-reperfusion, heart tissues were double-stained with Evans blue and TTC to determine the infarct area, AAR (indicating the ischemic area), and normally perfused area of the LV (Fig 3A and 3B). The four groups were comparable in terms of ischemic size (Fig 3C). In WT mice, IPC significantly reduced the myocardial infarct size (Fig 3D). As we previously reported [30, 34], SOCS3-CKO mice without IPC exhibited a significantly reduced myocardial infarct size compared to WT mice without IPC (Fig 3D). In SOCS3-CKO mice, IPC substantially reduced the myocardial infarct size (by approximately 90%) after I/R (Fig 3D).

### Myocardial STAT3 activation by ischemic preconditioning in infarct hearts after ischemia reperfusion in WT mice and SOCS3-CKO mice

**Fig 4:**
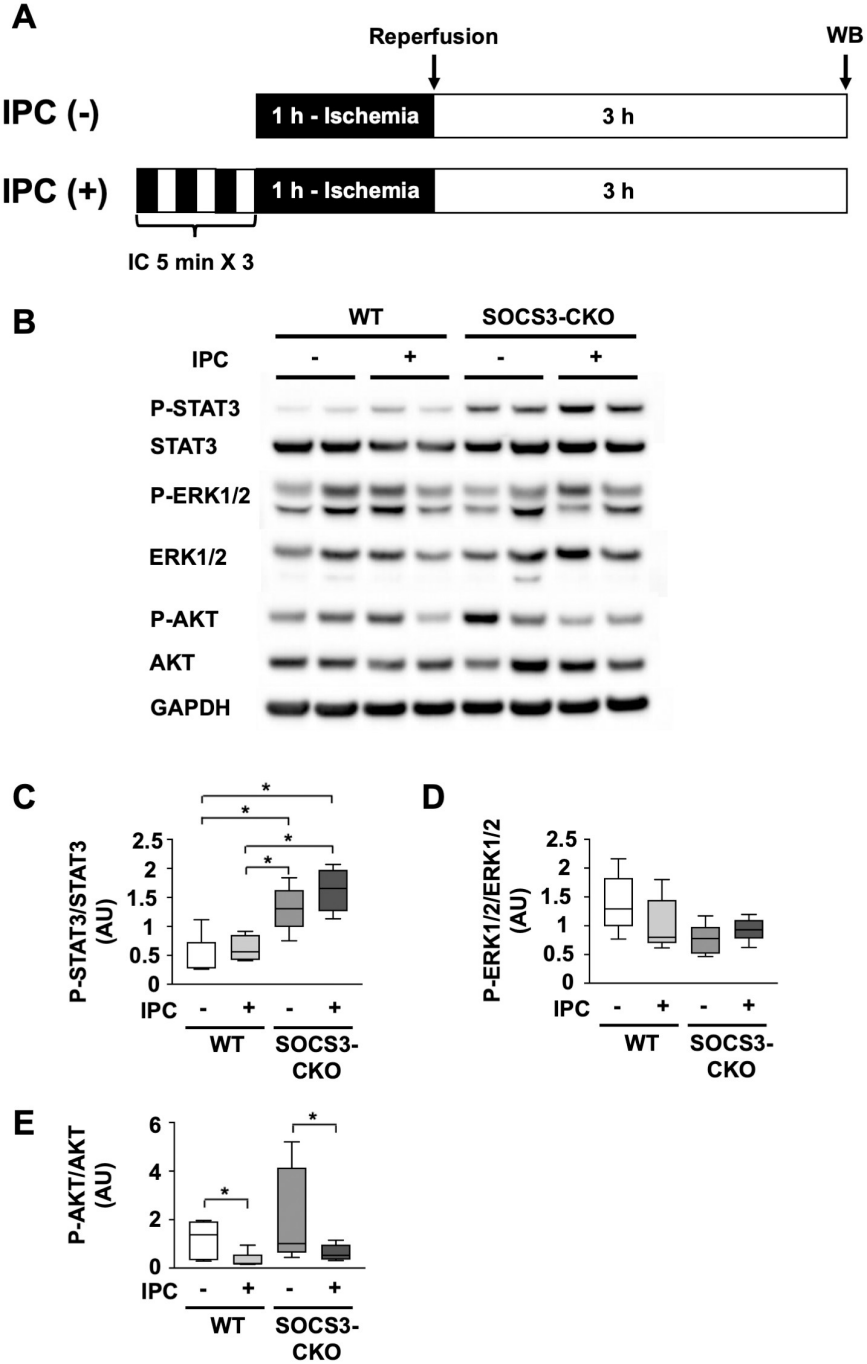
Activation of cardioprotective signaling molecules by ischemic preconditioning in myocardial I/R injury. (**A)** Schematic illustration depicting the experimental protocol. IPC was performed with three cycles of 5-min ischemia and 5-min reperfusion. The LAD coronary artery was occluded for 1 h, and then reperfused for 3 h. **(B)** Total cell lysates were prepared from heart tissue obtained at 3 h after I/R from WT mice with or without IPC, and from SOCS3-CKO mice with or without IPC. Lysates were blotted with antibodies raised against phosphorylated STAT3 (P-STAT3), total STAT3, phosphorylated ERK1/2 (P-ERK1/2), total ERK1/2, phosphorylated AKT (P-AKT), total AKT, and GAPDH. (**C)**, **(D)**, and **(E)** Box-and-whisker plots represent quantitative differences in P-STAT3, P-ERK1/2, and P-AKT expression (n = 4–5 per group). *p < 0.05 (Wilcoxon rank-sum test). I/R, ischemia reperfusion; IPC, ischemic preconditioning; LAD, left anterior descending; AU, arbitrary units; GAPDH, glyceraldehyde 3-phosphate dehydrogenase.

We performed western blot analysis for phosphorylated STAT3, ERK1/2, and AKT in protein samples collected at 3 h after I/R, from WT and SOCS3-CKO mice, with or without IC (Fig 4A). We detected greater STAT3 phosphorylation after IPC in the infarct hearts from SOCS3-CKO mice compared to WT mice (Fig 4B and 4C). Additionally, in SOCS3-CKO mice, the mean STAT3 phosphorylation level was higher with IPC than without IPC, although this difference was not significant (Fig 4B and 4C). ERK1/2 phosphorylation was comparable among the four groups (Fig 4B and 4D). Among both WT and SOCS3-CKO mice, AKT phosphorylation was lower with IPC than without IPC. (Fig 4B and 4E).

### Augmented expressions of anti-apoptotic genes and SAFE pathway-related genes in SOCS3-CKO mice following ischemic conditioning

**Fig 5:**
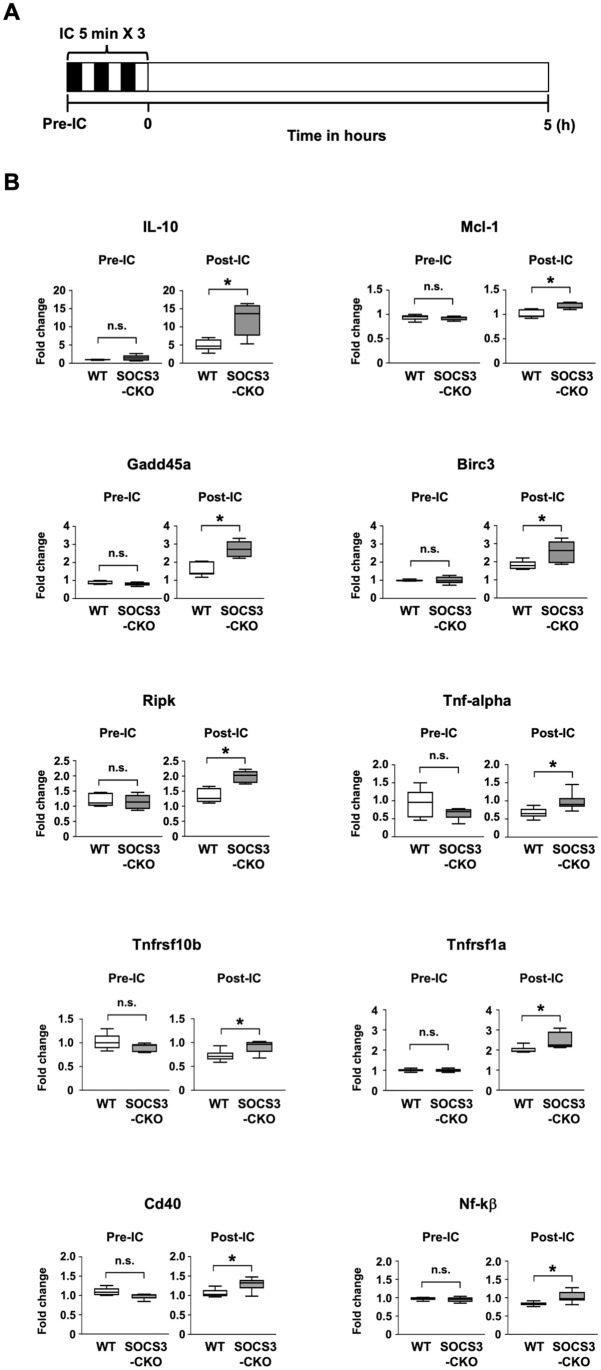
Expressions of apoptosis-related genes in heart tissue before and after ischemic conditioning. We prepared mRNA from heart tissue of WT or SOCS3-CKO mice, obtained pre-IC and at 5 h after IC. This mRNA was subjected to real-time PCR analysis. (**A**) Schematic illustration depicting the experimental protocol. **(B)** Values are expressed as fold change relative to the values from WT mice of pre-IC (n = 5–6 for each group). *p < 0.05 (Wilcoxon rank-sum test). Il-10 (interleukin-10), Mcl-1 (Myeloid cell leukemia sequence 1), Gadd45 (Growth arrest and DNA-damage-inducible 45 alpha), Birc3 (Baculoviral IAP repeat-containing 3), Ripk1 (Receptor (TNFRSF)-interacting serine-threonine kinase 1), Tnf-alpha (Tumor necrosis factor-alpha), Tnfrsf1a (Tumor necrosis factor receptor superfamily, member 1a), Tnfrsf10b (Tumor necrosis factor receptor superfamily, member 10b), Cd40 (CD40 antigen), and Nfkb1 (Nuclear factor of kappa light polypeptide gene enhancer in B-cells 1).

To elucidate the events downstream of STAT3, we performed real-time PCR array analysis for apoptosis-related genes in heart tissue before and after IC. Before IC, the expressions of most genes were comparable between SOCS3-CKO mice and WT mice (Fig 5). Only three genes showed significantly altered expression in heart tissues from SOCS3-CKO mice compared to WT mice: Card10 (Caspase recruitment domain family, member 10), Cideb (Cell death-inducing DNA fragmentation factor, alpha subunit-like effector B), and Diablo (Diablo homolog, Drosophila) (S3 Fig). At 5 h after IC, compared to WT mice, SOCS3-CKO mice exhibited significantly greater heart expression of several anti-apoptotic genes, including Il-10 (interleukin-10), Mcl-1 (Myeloid cell leukemia sequence 1), Gadd45 (Growth arrest and DNA-damage-inducible 45 alpha), Birc3 (Baculoviral IAP repeat-containing 3), and Ripk1 (Receptor (TNFRSF)-interacting serine-threonine kinase 1) (Fig 5). Additionally, compared to WT mice, the post-IC heart tissue of SOCS3-CKO mice showed significantly greater expressions of SAFE pathway-related genes, including STAT3-related genes and TNF-alpha-related genes—for example, Il-10, Mcl-1, Gadd45, Tnf-alpha (Tumor necrosis factor-alpha), Tnfrsf1a (Tumor necrosis factor receptor superfamily, member 1a), Tnfrsf10b (Tumor necrosis factor receptor superfamily, member 10b), Cd40 (CD40 antigen), and Nfkb1 (Nuclear factor of kappa light polypeptide gene enhancer in B-cells 1) (Fig 5).

We also evaluated RISK pathway-related genes in heart tissue from the pre-IC and 5 h after IC time-points, using PCR array analysis for apoptosis-related genes. RISK pathway-related genes include target molecules of ERK1/2 and AKT, such as Bax (Bcl2-associated X protein), Bcl-2 (B-cell leukemia/lymphoma 2), Bad (BCL2-associated agonist of cell death), Bcl-xl (Bcl2-like 1), Casp9 (Caspase 9), Dapk1 (Death associated protein kinase 1), Mcl-1 (Myeloid cell leukemia sequence 1). At 5 h after IC, only the Mcl-1 gene, which is also a SFAE-related gene, exhibited significantly altered expression in heart tissue from SOCS3-CKO mice compared to WT mice (S4 Fig).

### Rapid increase of circulating erythropoietin level after ischemic conditioning

**Fig 6:**
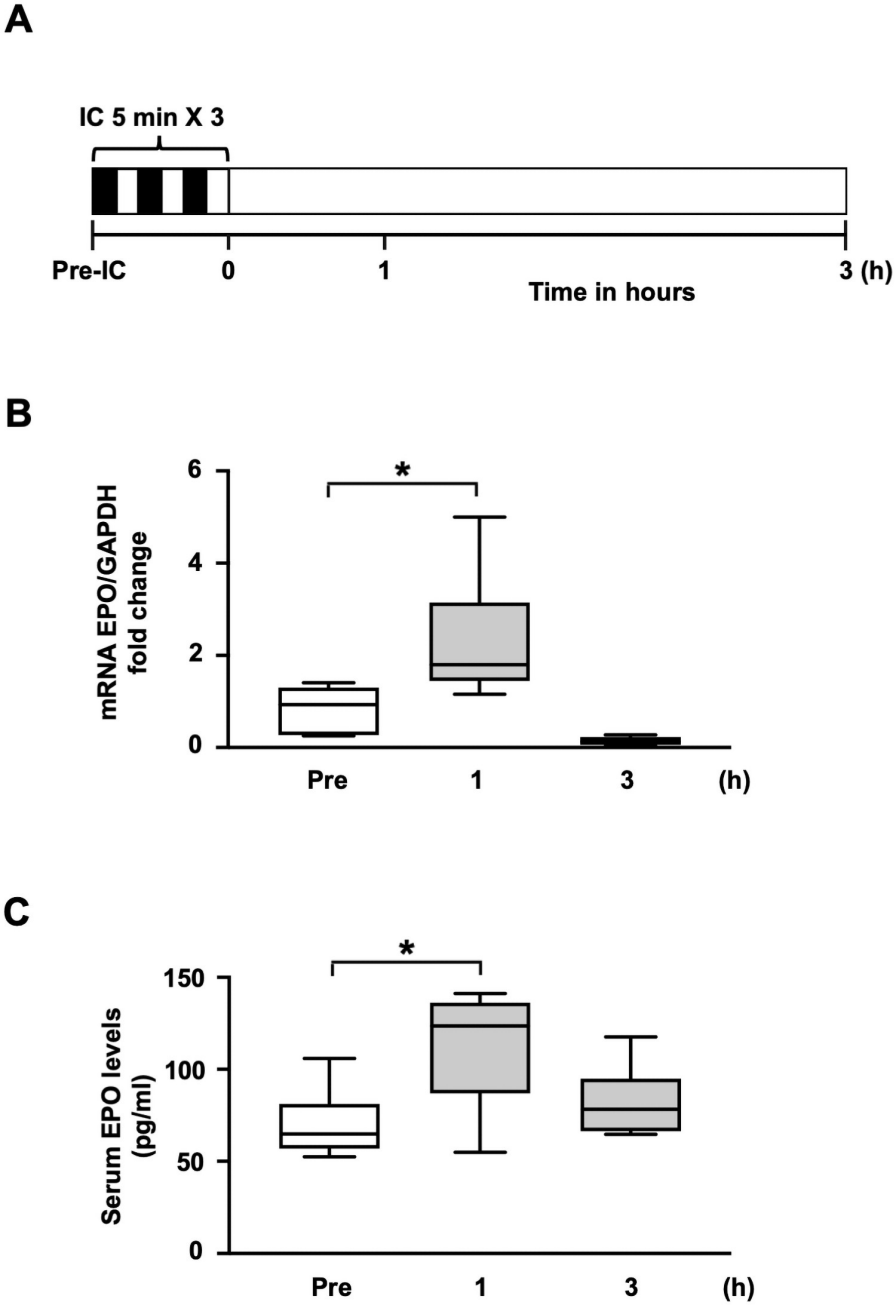
Rapid increase of circulating erythropoietin level after ischemic conditioning. (**A)** Schematic illustration depicting the experimental protocol. IC was performed with three cycles of 5-min ischemia and 5-min reperfusion. Kidneys were collected at 1 h or 3 h after cardiac IC. Serum was collected at 1 h or 3 h after cardiac IC. (**B)** EPO expression in the kidney after IC, analyzed by real-time PCR (n = 6–8 per group). Values are normalized to GAPDH, and expressed as a fold change from pre-IC values. *P<0.05 (Dunn’s test). (**C)** Serum levels of EPO in mice after IC at the indicated time-points, determined by an ELISA (n = 6–7 per group). *P<0.05. (Dunn’s test). EPO, erythropoietin; IC, ischemic conditioning; PCR, polymerase chain reaction; ELISA, enzyme-linked immunosorbent assay; GAPDH, glyceraldehyde 3-phosphate dehydrogenase.

We previously reported that remote IPC via repeated transient limb ischemia rapidly activates the renal hypoxia-inducible factor-1α (HIF1α)-EPO pathway, leading to EPO production and release from the kidney, and that this released EPO activates cardioprotective signals, including STAT3, thus reducing infarct size [38]. Based on these previous findings, we performed real-time PCR analysis and ELISA to examine whether myocardial IPC alone would induce EPO expression in the kidney (Fig 6A). Our results showed that renal EPO mRNA expression was promptly and significantly increased at 1 h after myocardial IC alone in WT mice (Fig 6B). ELISA revealed that circulating EPO levels were rapidly increased at 1 h after myocardial IC alone (Fig 6C).

## Discussion

In the present study, we have first investigated the effects of myocardial SOCS3 deficiency on IPC-mediated cardioprotection during myocardial I/R injury. In WT mice, IC alone transiently activated myocardial STAT3, and correspondingly induced SOCS3 expression. IC-induced STAT3 activation was significantly greater and more sustained in SOCS3-CKO mice than in WT mice. Following I/R, IPC substantially reduced infarct size and significantly enhanced STAT3 phosphorylation in SOCS3-CKO mice compared to in WT mice. Moreover, in WT mice, myocardial IC alone rapidly induced mRNA expression of the STAT3-activating cytokine EPO in the kidney, and increased the circulating EPO level. Thus, myocardial SOCS3 deficiency and IPC exerted synergistic effects in the prevention of myocardial injury after I/R. Our present results suggest that myocardial SOCS3 is a potent inhibitor of IPC-induced cardioprotection, and that myocardial SOCS3 inhibition augments IPC-mediated cardioprotection during I/R.

### Advantage of using myocardial-specific SOCS3-deficient mice

The STAT3 signaling pathway plays important roles in myocardial hypertrophy, apoptosis, and fibrosis [12–14]. αMHC-Cre-mediated SOCS3 ablation can occur as early as in the late stage of myocardium development [39]. Therefore, to clarify the developmental effects of αMHC-Cre-mediated SOCS3 ablation in the heart, we assessed the ratio of body weight-to-heart weight, performed histological analyses with hematoxylin and eosin and Mallory-Azan staining, and performed echocardiography using intact hearts from WT and SOCS3-CKO mice. The ratio of body weight-to-heart weight was comparable between SOCS3-CKO and WT mice (S1 Fig). Compared to hearts from WT mice, the hearts from SOCS3-CKO mice showed no evidence of necrosis, cardiac fibrosis, or hypertrophy (S1 Fig). Echocardiography revealed that left ventricular thickness, chamber size, and systolic function were comparable between SOCS3-CKO mice and WT mice (S2 Fig). Thus, global cardiac structure and function in intact hearts were comparable between SOCS3-CKO and WT mice.

Many lines of evidences indicate that the STAT3 signaling pathway has potent pro-survival activity against myocardial injury during acute MI [12–17, 40]. STAT3 is activated by different types of IC—including IPC, ischemic postconditioning, and remote IPC [16, 19, 38, 41]. This suggests that STAT3 activation is a common signal of cardioprotection through IC. The causal involvement of STAT3 in IPC-mediated cardioprotection during I/R has been demonstrated in prior studies using myocardial STAT3-deficient mice [11, 19, 22] or the pharmacological JAK inhibitor AG490 [11, 24, 41]. On the other hand, in our present study we showed the causal role of myocardial STAT3 in IPC-mediated cardioprotection during I/R using myocardial SOCS3-deficient mice, which exhibited augmented and prolonged endogenous myocardial STAT3 activation. The SOCS3 promoter contains a functionally important STAT3-binding element [42], and SOCS3 binds to JAK through the receptors of STAT3-activating cytokines, including EPO and G-CSF, thus inhibiting JAK kinase activity [43, 44]. This suggests that SOCS3 is a highly specific negative-feedback regulator of the STAT3 signaling pathway. Therefore, an advantage of using SOCS3-CKO mice is that endogenous STAT3 activation in cardiomyocytes is specifically enhanced in response to intervention, such as IC or pathological stimuli. In fact, in the present study, compared to WT mice, SOCS3-CKO mice exhibited enhanced STAT3 activation in the heart in response to IC alone or IPC plus I/R.

### SOCS3 is an ischemic conditioning-inducible potent inhibitor of IPC-mediated cardioprotection

Negative-feedback regulation through SOCS3 tightly regulates the duration and intensity of STAT3-activating cytokine activities [25–27]. As an intrinsic STAT3 inhibitor, SOCS3 has three prominent features. First, as mentioned above, SOCS3 is a highly specific negative-feedback regulator of the STAT3 signaling pathway. Second, SOCS3 is inducible protein by STAT3-activating cytokines or myocardial injury stress. We previously reported that SOCS3 is promptly induced by cardioprotective EPO, G-CSF, and gp130 cytokines, including cardiotrophin1 and leukemia inhibitory factor [28, 33, 43]. We have also demonstrated that SOCS3 is rapidly induced during pressure overload, viral myocarditis, post-MI ventricular remodeling, and I/R injury in mice [30–34]. In our present study, we observed prompt SOCS3 induction in heart tissue after IC, which was induced by a brief episode of nonlethal myocardial ischemia and reperfusion. Third, SOCS3 is a very potent inhibitor of the STAT3 signaling pathway. We previously demonstrated that forced SOCS3 expression completely suppressed gp130-mediated STAT3 activation and survival of cardiomyocytes [33], and that the hearts of SOCS3 transgenic mice exhibited markedly increased susceptibility to viral infection [32]. Our present results showed that IPC markedly reduced myocardial infarct size after I/R in SOCS3-CKO mice. Overall, our data indicate that SOCS3 is an IC-inducible potent inhibitor of IPC-mediated cardioprotection.

### Important role of renal erythropoietin in cardioprotection via cardiac IPC during myocardial ischemia reperfusion injury

**Fig 7:**
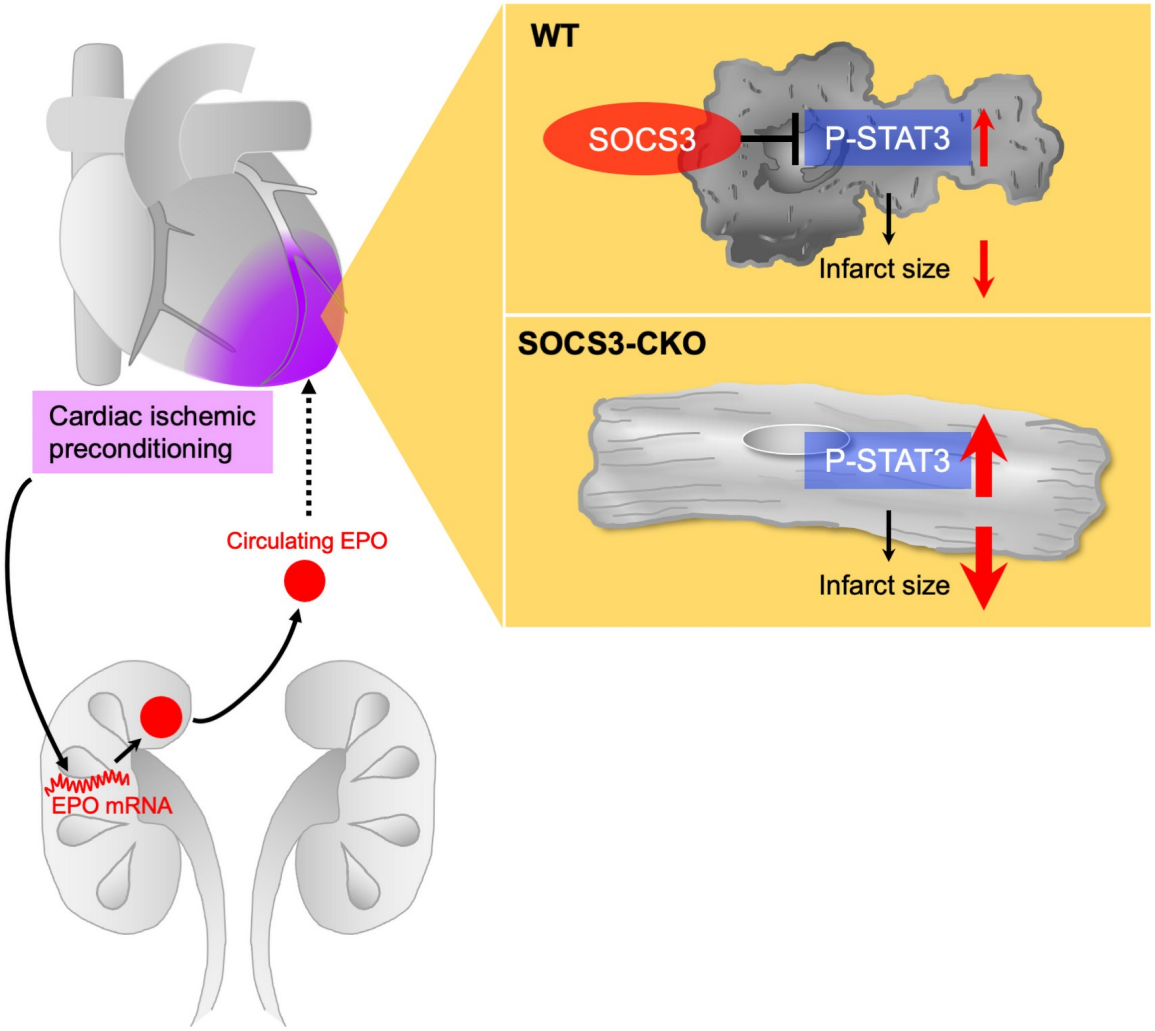
Summary and model of synergistic myocardial infarct size reduction by IPC in cardiac-specific SOCS3-deficient mice. Cardiac IPC, induced by repeated non-lethal ischemia and reperfusion of coronary artery, activates the renal EPO pathway. EPO is produced and released, and binds to myocardial EPO receptors, thus possibly activating the cardioprotective STAT3 signaling pathway. In the myocardium of WT mice, cardiac IPC-induced STAT3 activation was suppressed by SOCS3. In contrast, in the myocardium of SOCS3-CKO mice, cardiac IPC-induced STAT3 activation was enhanced and prolonged, thus synergistically preventing myocardial infarction after I/R. IPC, ischemic preconditioning; EPO, erythropoietin; WT, wild-type; I/R, ischemia reperfusion.

We previously proposed a novel remote IPC mechanism through the kidney [38]. Briefly, repeated transient limb ischemia rapidly induces renal HIF1α expression, leading to EPO production and release from the kidney. Then this released EPO activates cardioprotective signals, including STAT3, thus reducing infarct size [38]. Based on previous findings, in our present study, we expected that the renal HIF1α-EPO pathway would be involved in the mechanism of cardioprotection via cardiac IPC. Fig 7 presents a summary and model of the findings of our current study. Cardiac IPC, induced by repeated occlusion and release of coronary artery, activates the renal HIF1α-EPO pathway, leading to EPO production and release, which activates the myocardial STAT3 signaling pathway. In the myocardium of WT mice, cardiac IPC-induced STAT3 activation was suppressed by SOCS3. In contrast, in the myocardium of SOCS3-CKO mice, cardiac IPC-induced STAT3 activation was enhanced and prolonged, and synergistically prevented myocardial injury after I/R. Thus, renal EPO may play an important role in cardiac IPC-induced cardioprotection during I/R injury.

### Combination of SOCS3 inhibition and STAT3 activation is a novel strategy for myocardial ischemia reperfusion injury

Promising experimental results in animal models support a real cardioprotective effect of STAT3-activating cytokines, including EPO and G-CSF; however, clinical trials have not yet demonstrated benefits in clinical settings [45–48]. Importantly, the state of the heart critically differs between animal models and real acute MI patients. While the animals used for experiments are young and healthy, and have intact hearts, most acute MI patients are older and have cardiovascular diseases, such as hypertension and coronary artery diseases. We previously demonstrated that myocardial stress, such as pressure overload and ischemia, rapidly and strongly induce SOCS3 [30, 31, 33]. Moreover, in cytokine therapy using EPO or G-CSF, SOCS3 is strongly induced by cytokine administration itself, suggesting that patients undergoing cytokine therapy may exhibit upregulated cardiac SOCS3 expression. Furthermore, we previously demonstrated that SOCS3 confers potent resistance to cytokines (e.g., cardiotrophin-1, leptin, and interferon) that utilize the STAT3 pathway [49–51]. Therefore, SOCS3 may reduce the effects of cytokine therapy in patients with acute MI. In fact, our present study revealed that myocardial SOCS3 deletion markedly augmented the IPC-induced cardioprotective effect during myocardial injury after I/R. Together, the previous and present findings suggest that a combination of SOCS3 inhibition and STAT3 activation may be a novel strategy for acute MI after I/R.

There is currently no clinical strategy for SOCS3 suppression. In animal studies, SOCS3 has been inhibited using adeno-associated virus 9 (AAV9)-mediated RNA interference or administration of the anti-diabetic drug pioglitazone [52, 53]. Using a rat model of dilated heart failure, Gao et al. reported that SOCS3 gene silencing using AAV9-SOCS3 siRNA led to significant reductions of myocardial fibrosis and inflammatory response, and improved heart function [52]. Collino et al. reported that pioglitazone administration reduced SOCS3 expression in the liver of rats fed a high-cholesterol fructose diet [53]. However, the mechanism through which pioglitazone inhibits SOCS3 expression is unclear. Thus, further studies are needed to examine how pioglitazone inhibits SOCS3 expression, and whether pioglitazone augments STAT3 activity resulting from SOCS3 inhibition.

We previously reported the molecular mechanism through which SOCS1 and SOCS3 inhibit JAK kinase activity [28, 29]. The kinase inhibitory region of SOCS1 and SOCS3 targets the activation loop of the JAK kinase domain with high specificity, thereby blocking any subsequent phosphorylation. We previously proposed that SOCS1 and SOCS3 inhibit JAK kinase activity via binding in the activation loop as a pseudosubstrate [28, 29] and this was confirmed by X-ray crystal structure analysis [54]. Therefore, small-molecule compounds that inhibit the binding of JAK kinase activation loops to the SOCS3 kinase inhibitory region may be clinically useful for SOCS3 inhibition.

### Study limitations

In this study, it considered that STAT3 phosphorylation is a key target for IPC and SOCS3 inhibition to induce cardioprotection during myocardial I/R injury. Therefore, it will be important to confirm whether cardioprotection during myocardial IR injury is mediated by myocardial STAT3 phosphorylation induced by IPC and SOCS3 inhibition. To this end, future studies should investigate whether cardiac STAT3 inhibition abrogates the cardioprotective effect of IPC and SOCS3 ablation during myocardial IR injury. Optimal experiments would be performed using cardiac-specific STAT3 and SOCS3 double-knockout mice.

## Conclusion

Our present study showed that cardiac IC alone transiently activated myocardial STAT3 and correspondingly induced SOCS3 expression in WT mice, and that IC-induced STAT3 activation was significantly greater and more sustained in SOCS3-CKO mice than in WT mice. Following I/R, IPC substantially reduced myocardial infarct size and significantly enhanced STAT3 phosphorylation in SOCS3-CKO mice compared to in WT mice. Moreover, myocardial IC alone rapidly induced renal EPO mRNA expression and increased the circulating EPO level in WT mice. Our present results suggest that myocardial SOCS3 is a potent inhibitor of IPC-induced cardioprotection and that myocardial SOCS3 inhibition augment IPC-mediated cardioprotection during I/R. We propose that the combination of myocardial SOCS3 downregulation and STAT3 activation via IPC may be a novel strategy for reducing myocardial injury after I/R.

## Supporting information

S1 Fig Histological staining and ratio of body weight-to-heart weight in WT and SOCS3-CKO mice. (**A**) Graph shows the ratio of heart weight-to-body weight based on intact hearts (n = 5 per group, Wilcoxon rank-sum test). (**B**) Sections from intact hearts of WT mice and SOCS3-CKO mice were stained with hematoxylin and eosin and Mallory-Azan (n = 5 per group).
(TIF)

S2 Fig Echocardiography of WT and SOCS3-CKO mice. Echocardiography was performed in intact WT mice and SOCS3-cKO mice (n = 11–14 per group, Wilcoxon rank sum test). IVST, interventricular septum thickness; PWT, posterior left ventricular wall thickness; LVEDD, left ventricular end-diastolic diameter; LVESD, left ventricular end-systolic diameter; FS, fractional shortening.
(TIF)

S3 Fig Expression of apoptosis-related genes in heart tissue before and after ischemic conditioning. We prepared mRNA from heart tissue of WT or SOCS3-CKO mice, obtained pre-IC and 5 h after IC. This mRNA was subjected to real-time PCR analysis. Values are expressed as fold change relative to the values from WT mice of pre-IC (n = 5–6 for each group). *p < 0.05 (Wilcoxon rank-sum test). Card10 (Caspase recruitment domain family, member 10), Cideb (Cell death-inducing DNA fragmentation factor, alpha subunit-like effector B), Diablo (Diablo homolog, Drosophila).
(TIF)

S4 Fig Expression of apoptosis-related molecules involved in the RISK pathway. We prepared mRNA from heart tissue of WT or SOCS3-CKO mice, obtained pre-IC or 5 h after IC. This mRNA was subjected to real-time PCR analysis. Values are expressed as fold change relative to the values from WT mice of pre-IC (n = 5–6 for each group). *p < 0.05 (Wilcoxon rank-sum test). Bax (Bcl2-associated X protein), Bcl-2 (B-cell leukemia/lymphoma 2), Bad (BCL2-associated agonist of cell death), Bcl-xl (Bcl2-like 1), Casp9 (Caspase 9), Dapk1 (Death associated protein kinase 1), Mcl-1 (Myeloid cell leukemia sequence 1).
(TIF)

S5 Fig Full unedited blots for Fig 1. Blots were probed using antibodies against tyrosine- phosphorylated STAT3 (P-STAT3), STAT3, phosphorylated AKT (P-AKT), AKT, phosphorylated ERK (P-ERK1/2), and GAPDH.
(TIF)

S6 Fig Full unedited blots for Fig 2. Blots were probed using antibodies against tyrosine- phosphorylated STAT3 (P-STAT3), STAT3, phosphorylated AKT (P-AKT), AKT, phosphorylated ERK (P-ERK1/2), and GAPDH.
(TIF)

S7 Fig Full unedited blots for Fig 4. Blots were probed using antibodies against tyrosine- phosphorylated STAT3 (P-STAT3), STAT3, phosphorylated AKT (P-AKT), AKT, phosphorylated ERK (P-ERK1/2), and GAPDH.
(TIF)

## References

[1] Hausenloy DJ, Botker HE, Engstrom T, Erlinge D, Heusch G, Ibanez B, et al. Targeting reperfusion injury in patients with ST-segment elevation myocardial infarction: trials and tribulations. Eur Heart J. 2017;38(13):935–41. Epub 2016/04/28. doi: 10.1093/eurheartj/ehw145 . 27118196 PMC5381598

[2] Hausenloy DJ, Yellon DM. Ischaemic conditioning and reperfusion injury. Nat Rev Cardiol. 2016;13(4):193–209. Epub 2016/02/05. doi: 10.1038/nrcardio.2016.5 . 26843289

[3] Heusch G. Myocardial ischaemia-reperfusion injury and cardioprotection in perspective. Nat Rev Cardiol. 2020;17(12):773–89. Epub 2020/07/06. doi: 10.1038/s41569-020-0403-y . 32620851

[4] Heusch G, Gersh BJ. The pathophysiology of acute myocardial infarction and strategies of protection beyond reperfusion: a continual challenge. Eur Heart J. 2017;38(11):774–84. Epub 2016/06/30. doi: 10.1093/eurheartj/ehw224 . 27354052

[5] Kloner RA, Brown DA, Csete M, Dai W, Downey JM, Gottlieb RA, et al. New and revisited approaches to preserving the reperfused myocardium. Nat Rev Cardiol. 2017;14(11):679–93. Epub 2017/07/28. doi: 10.1038/nrcardio.2017.102 . 28748958 PMC5991096

[6] Lecour S, Bøtker HE, Condorelli G, Davidson SM, Garcia-Dorado D, Engel FB, et al. ESC working group cellular biology of the heart: position paper: improving the preclinical assessment of novel cardioprotective therapies. Cardiovasc Res. 2014;104(3):399–411. Epub 2014/10/26. doi: 10.1093/cvr/cvu225 . 25344369 PMC4242141

[7] Murry CE, Jennings RB, Reimer KA. Preconditioning with ischemia: a delay of lethal cell injury in ischemic myocardium. Circulation. 1986;74(5):1124–36. Epub 1986/11/01. doi: 10.1161/01.cir.74.5.1124 . 3769170

[8] Hausenloy DJ, Yellon DM. The therapeutic potential of ischemic conditioning: an update. Nat Rev Cardiol. 2011;8(11):619–29. Epub 2011/06/22. doi: 10.1038/nrcardio.2011.85 . 21691310

[9] Heusch G, Rassaf T. Time to Give Up on Cardioprotection? A Critical Appraisal of Clinical Studies on Ischemic Pre-, Post-, and Remote Conditioning. Circ Res. 2016;119(5):676–95. Epub 2016/08/20. doi: 10.1161/CIRCRESAHA.116.308736 . 27539973

[10] Minamino T. Cardioprotection from ischemia/reperfusion injury: basic and translational research. Circ J. 2012;76(5):1074–82. Epub 2012/04/17. doi: 10.1253/circj.cj-12-0132 . 22504127

[11] Bolli R, Stein AB, Guo Y, Wang OL, Rokosh G, Dawn B, et al. A murine model of inducible, cardiac-specific deletion of STAT3: its use to determine the role of STAT3 in the upregulation of cardioprotective proteins by ischemic preconditioning. J Mol Cell Cardiol. 2011;50(4):589–97. Epub 2011/01/13. doi: 10.1016/j.yjmcc.2011.01.002 . 21223971

[12] Fischer P, Hilfiker-Kleiner D. Survival pathways in hypertrophy and heart failure: the gp130-STAT3 axis. Basic Res Cardiol. 2007;102(4):279–97. Epub 2007/05/29. doi: 10.1007/s00395-007-0658-z . 17530315

[13] Harhous Z, Booz GW, Ovize M, Bidaux G, Kurdi M. An Update on the Multifaceted Roles of STAT3 in the Heart. Front Cardiovasc Med. 2019;6:150. Epub 2019/11/12. doi: 10.3389/fcvm.2019.00150 . 31709266 PMC6823716

[14] Hilfiker-Kleiner D, Hilfiker A, Fuchs M, Kaminski K, Schaefer A, Schieffer B, et al. Signal transducer and activator of transcription 3 is required for myocardial capillary growth, control of interstitial matrix deposition, and heart protection from ischemic injury. Circ Res. 2004;95(2):187–95. Epub 2004/06/12. doi: 10.1161/01.RES.0000134921.50377.61 . 15192020

[15] Kleinbongard P, Skyschally A, Gent S, Pesch M, Heusch G. STAT3 as a common signal of ischemic conditioning: a lesson on "rigor and reproducibility" in preclinical studies on cardioprotection. Basic Res Cardiol. 2018;113(1):3. Epub 2017/11/22. doi: 10.1007/s00395-017-0660-z . 29159507

[16] Obana M, Miyamoto K, Murasawa S, Iwakura T, Hayama A, Yamashita T, et al. Therapeutic administration of IL-11 exhibits the postconditioning effects against ischemia-reperfusion injury via STAT3 in the heart. Am J Physiol Heart Circ Physiol. 2012;303(5):H569–77. Epub 2012/06/19. doi: 10.1152/ajpheart.00060.2012 . 22707562

[17] Zouein FA, Altara R, Chen Q, Lesnefsky EJ, Kurdi M, Booz GW. Pivotal Importance of STAT3 in Protecting the Heart from Acute and Chronic Stress: New Advancement and Unresolved Issues. Front Cardiovasc Med. 2015;2:36. Epub 2015/12/15. doi: 10.3389/fcvm.2015.00036 . 26664907 PMC4671345

[18] Lecour S. Activation of the protective Survivor Activating Factor Enhancement (SAFE) pathway against reperfusion injury: Does it go beyond the RISK pathway? J Mol Cell Cardiol. 2009;47(1):32–40. Epub 2009/04/07. doi: 10.1016/j.yjmcc.2009.03.019 . 19344728

[19] Boengler K, Buechert A, Heinen Y, Roeskes C, Hilfiker-Kleiner D, Heusch G, et al. Cardioprotection by ischemic postconditioning is lost in aged and STAT3-deficient mice. Circ Res. 2008;102(1):131–5. Epub 2007/10/31. doi: 10.1161/CIRCRESAHA.107.164699 . 17967780

[20] Hadebe N, Cour M, Lecour S. The SAFE pathway for cardioprotection: is this a promising target? Basic Res Cardiol. 2018;113(2):9. Epub 2018/01/18. doi: 10.1007/s00395-018-0670-5 . 29335904

[21] Heusch G. Molecular basis of cardioprotection: signal transduction in ischemic pre-, post-, and remote conditioning. Circ Res. 2015;116(4):674–99. Epub 2015/02/14. doi: 10.1161/CIRCRESAHA.116.305348 . 25677517

[22] Smith RM, Suleman N, Lacerda L, Opie LH, Akira S, Chien KR, et al. Genetic depletion of cardiac myocyte STAT-3 abolishes classical preconditioning. Cardiovasc Res. 2004;63(4):611–6. Epub 2004/08/13. doi: 10.1016/j.cardiores.2004.06.019 . 15306216

[23] Suleman N, Somers S, Smith R, Opie LH, Lecour SC. Dual activation of STAT-3 and Akt is required during the trigger phase of ischaemic preconditioning. Cardiovasc Res. 2008;79(1):127–33. Epub 2008/03/15. doi: 10.1093/cvr/cvn067 . 18339648

[24] Gent S, Skyschally A, Kleinbongard P, Heusch G. Ischemic preconditioning in pigs: a causal role for signal transducer and activator of transcription 3. Am J Physiol Heart Circ Physiol. 2017;312(3):H478–h84. Epub 2017/01/01. doi: 10.1152/ajpheart.00749.2016 . 28039203

[25] Naka T, Narazaki M, Hirata M, Matsumoto T, Minamoto S, Aono A, et al. Structure and function of a new STAT-induced STAT inhibitor. Nature. 1997;387(6636):924–9. Epub 1997/06/26. doi: 10.1038/43219 . 9202127

[26] Yasukawa H, Nagata T, Oba T, Imaizumi T. SOCS3: A novel therapeutic target for cardioprotection. Jakstat. 2012;1(4):234–40. Epub 2013/09/24. doi: 10.4161/jkst.22435 . 24058778 PMC3670279

[27] Yasukawa H, Sasaki A, Yoshimura A. Negative regulation of cytokine signaling pathways. Annu Rev Immunol. 2000;18:143–64. Epub 2000/06/03. doi: 10.1146/annurev.immunol.18.1.143 . 10837055

[28] Sasaki A, Yasukawa H, Suzuki A, Kamizono S, Syoda T, Kinjyo I, et al. Cytokine-inducible SH2 protein-3 (CIS3/SOCS3) inhibits Janus tyrosine kinase by binding through the N-terminal kinase inhibitory region as well as SH2 domain. Genes Cells. 1999;4(6):339–51. Epub 1999/07/28. doi: 10.1046/j.1365-2443.1999.00263.x . 10421843

[29] Yasukawa H, Misawa H, Sakamoto H, Masuhara M, Sasaki A, Wakioka T, et al. The JAK-binding protein JAB inhibits Janus tyrosine kinase activity through binding in the activation loop. Embo j. 1999;18(5):1309–20. Epub 1999/03/04. doi: 10.1093/emboj/18.5.1309 . 10064597 PMC1171221

[30] Nagata T, Yasukawa H, Kyogoku S, Oba T, Takahashi J, Nohara S, et al. Cardiac-Specific SOCS3 Deletion Prevents In Vivo Myocardial Ischemia Reperfusion Injury through Sustained Activation of Cardioprotective Signaling Molecules. PLoS One. 2015;10(5):e0127942. Epub 2015/05/27. doi: 10.1371/journal.pone.0127942 . 26010537 PMC4444323

[31] Oba T, Yasukawa H, Hoshijima M, Sasaki K, Futamata N, Fukui D, et al. Cardiac-specific deletion of SOCS-3 prevents development of left ventricular remodeling after acute myocardial infarction. J Am Coll Cardiol. 2012;59(9):838–52. Epub 2012/03/01. doi: 10.1016/j.jacc.2011.10.887 . 22361405

[32] Yajima T, Yasukawa H, Jeon ES, Xiong D, Dorner A, Iwatate M, et al. Innate defense mechanism against virus infection within the cardiac myocyte requiring gp130-STAT3 signaling. Circulation. 2006;114(22):2364–73. Epub 2006/11/15. doi: 10.1161/CIRCULATIONAHA.106.642454 . 17101849

[33] Yasukawa H, Hoshijima M, Gu Y, Nakamura T, Pradervand S, Hanada T, et al. Suppressor of cytokine signaling-3 is a biomechanical stress-inducible gene that suppresses gp130-mediated cardiac myocyte hypertrophy and survival pathways. J Clin Invest. 2001;108(10):1459–67. Epub 2001/11/21. doi: 10.1172/JCI13939 . 11714737 PMC209425

[34] Yasukawa H, Yajima T, Duplain H, Iwatate M, Kido M, Hoshijima M, et al. The suppressor of cytokine signaling-1 (SOCS1) is a novel therapeutic target for enterovirus-induced cardiac injury. J Clin Invest. 2003;111(4):469–78. Epub 2003/02/18. doi: 10.1172/JCI16491 . 12588885 PMC151924

[35] Yasukawa H, Ohishi M, Mori H, Murakami M, Chinen T, Aki D, et al. IL-6 induces an anti-inflammatory response in the absence of SOCS3 in macrophages. Nat Immunol. 2003;4(6):551–6. Epub 2003/05/20. doi: 10.1038/ni938 . 12754507

[36] Agah R, Frenkel PA, French BA, Michael LH, Overbeek PA, Schneider MD. Gene recombination in postmitotic cells. Targeted expression of Cre recombinase provokes cardiac-restricted, site-specific rearrangement in adult ventricular muscle in vivo. J Clin Invest. 1997;100(1):169–79. Epub 1997/07/01. doi: 10.1172/jci119509 . 9202069 PMC508177

[37] Takahashi J, Yamamoto M, Yasukawa H, Nohara S, Nagata T, Shimozono K, et al. Interleukin-22 Directly Activates Myocardial STAT3 (Signal Transducer and Activator of Transcription-3) Signaling Pathway and Prevents Myocardial Ischemia Reperfusion Injury. J Am Heart Assoc. 2020;9(8):e014814. Epub 2020/04/18. doi: 10.1161/JAHA.119.014814 . 32301368 PMC7428538

[38] Oba T, Yasukawa H, Nagata T, Kyogoku S, Minami T, Nishihara M, et al. Renal Nerve-Mediated Erythropoietin Release Confers Cardioprotection During Remote Ischemic Preconditioning. Circ J. 2015;79(7):1557–67. Epub 2015/04/03. doi: 10.1253/circj.CJ-14-1171 . 25833080

[39] Takahashi Y, Carpino N, Cross JC, Torres M, Parganas E, Ihle JN. SOCS3: an essential regulator of LIF receptor signaling in trophoblast giant cell differentiation. Embo j. 2003;22(3):372–84. Epub 2003/01/30. doi: 10.1093/emboj/cdg057 . 12554639 PMC140741

[40] Bolli R, Dawn B, Xuan YT. Role of the JAK-STAT pathway in protection against myocardial ischemia/reperfusion injury. Trends Cardiovasc Med. 2003;13(2):72–9. Epub 2003/02/15. doi: 10.1016/s1050-1738(02)00230-x . 12586443

[41] Butler KL, Huffman LC, Koch SE, Hahn HS, Gwathmey JK. STAT-3 activation is necessary for ischemic preconditioning in hypertrophied myocardium. Am J Physiol Heart Circ Physiol. 2006;291(2):H797–803. Epub 2006/03/28. doi: 10.1152/ajpheart.01334.2005 . 16565302

[42] Auernhammer CJ, Bousquet C, Melmed S. Autoregulation of pituitary corticotroph SOCS-3 expression: characterization of the murine SOCS-3 promoter. Proc Natl Acad Sci U S A. 1999;96(12):6964–9. Epub 1999/06/09. doi: 10.1073/pnas.96.12.6964 . 10359822 PMC22025

[43] Sasaki A, Yasukawa H, Shouda T, Kitamura T, Dikic I, Yoshimura A. CIS3/SOCS-3 suppresses erythropoietin (EPO) signaling by binding the EPO receptor and JAK2. J Biol Chem. 2000;275(38):29338–47. Epub 2000/07/07. doi: 10.1074/jbc.M003456200 . 10882725

[44] White CA, Nicola NA. SOCS3: An essential physiological inhibitor of signaling by interleukin-6 and G-CSF family cytokines. Jakstat. 2013;2(4):e25045. Epub 2014/01/15. doi: 10.4161/jkst.25045 . 24416642 PMC3876435

[45] Najjar SS, Rao SV, Melloni C, Raman SV, Povsic TJ, Melton L, et al. Intravenous erythropoietin in patients with ST-segment elevation myocardial infarction: REVEAL: a randomized controlled trial. Jama. 2011;305(18):1863–72. Epub 2011/05/12. doi: 10.1001/jama.2011.592 . 21558517 PMC3486644

[46] Ott I, Schulz S, Mehilli J, Fichtner S, Hadamitzky M, Hoppe K, et al. Erythropoietin in patients with acute ST-segment elevation myocardial infarction undergoing primary percutaneous coronary intervention: a randomized, double-blind trial. Circ Cardiovasc Interv. 2010;3(5):408–13. Epub 2010/08/26. doi: 10.1161/CIRCINTERVENTIONS.109.904425 . 20736448

[47] Steppich B, Hadamitzky M, Ibrahim T, Groha P, Schunkert H, Laugwitz KL, et al. Stem cell mobilisation by granulocyte-colony stimulating factor in patients with acute myocardial infarction. Long-term results of the REVIVAL-2 trial. Thromb Haemost. 2016;115(4):864–8. Epub 2016/01/23. doi: 10.1160/TH15-07-0589 . 26790705

[48] Zimmet H, Porapakkham P, Porapakkham P, Sata Y, Haas SJ, Itescu S, et al. Short- and long-term outcomes of intracoronary and endogenously mobilized bone marrow stem cells in the treatment of ST-segment elevation myocardial infarction: a meta-analysis of randomized control trials. Eur J Heart Fail. 2012;14(1):91–105. Epub 2011/11/09. doi: 10.1093/eurjhf/hfr148 . 22065869

[49] Hamanaka I, Saito Y, Yasukawa H, Kishimoto I, Kuwahara K, Miyamoto Y, et al. Induction of JAB/SOCS-1/SSI-1 and CIS3/SOCS-3/SSI-3 is involved in gp130 resistance in cardiovascular system in rat treated with cardiotrophin-1 in vivo. Circ Res. 2001;88(7):727–32. Epub 2001/04/17. doi: 10.1161/hh0701.088512 . 11304496

[50] Mori H, Hanada R, Hanada T, Aki D, Mashima R, Nishinakamura H, et al. Socs3 deficiency in the brain elevates leptin sensitivity and confers resistance to diet-induced obesity. Nat Med. 2004;10(7):739–43. Epub 2004/06/23. doi: 10.1038/nm1071 . 15208705

[51] Sakamoto H, Yasukawa H, Masuhara M, Tanimura S, Sasaki A, Yuge K, et al. A Janus kinase inhibitor, JAB, is an interferon-gamma-inducible gene and confers resistance to interferons. Blood. 1998;92(5):1668–76. Epub 1998/08/26. . 9716595

[52] Gao J, Guo Y, Chen Y, Zhou J, Liu Y, Su P. Adeno-associated virus 9-mediated RNA interference targeting SOCS3 alleviates diastolic heart failure in rats. Gene. 2019;697:11–8. Epub 2019/02/15. doi: 10.1016/j.gene.2019.01.044 . 30763670

[53] Collino M, Aragno M, Castiglia S, Miglio G, Tomasinelli C, Boccuzzi G, et al. Pioglitazone improves lipid and insulin levels in overweight rats on a high cholesterol and fructose diet by decreasing hepatic inflammation. Br J Pharmacol. 2010;160(8):1892–902. Epub 2010/03/18. doi: 10.1111/j.1476-5381.2010.00671.x . 20233219 PMC2958635

[54] Liau NPD, Laktyushin A, Lucet IS, Murphy JM, Yao S, Whitlock E, et al. The molecular basis of JAK/STAT inhibition by SOCS1. Nat Commun. 2018;9(1):1558. Epub 2018/04/21. doi: 10.1038/s41467-018-04013-1 . 29674694 PMC5908791

