# Supplementary figures and images for: SOCS3 deficiency in cardiomyocytes elevates sensitivity of ischemic preconditioning that synergistically ameliorates myocardial ischemia reperfusion injury
Source: PLoS One. 2021 Jul 22;16(7):e0254712. doi: 10.1371/journal.pone.0254712 (PMC8297769; doi:10.1371/journal.pone.0254712)

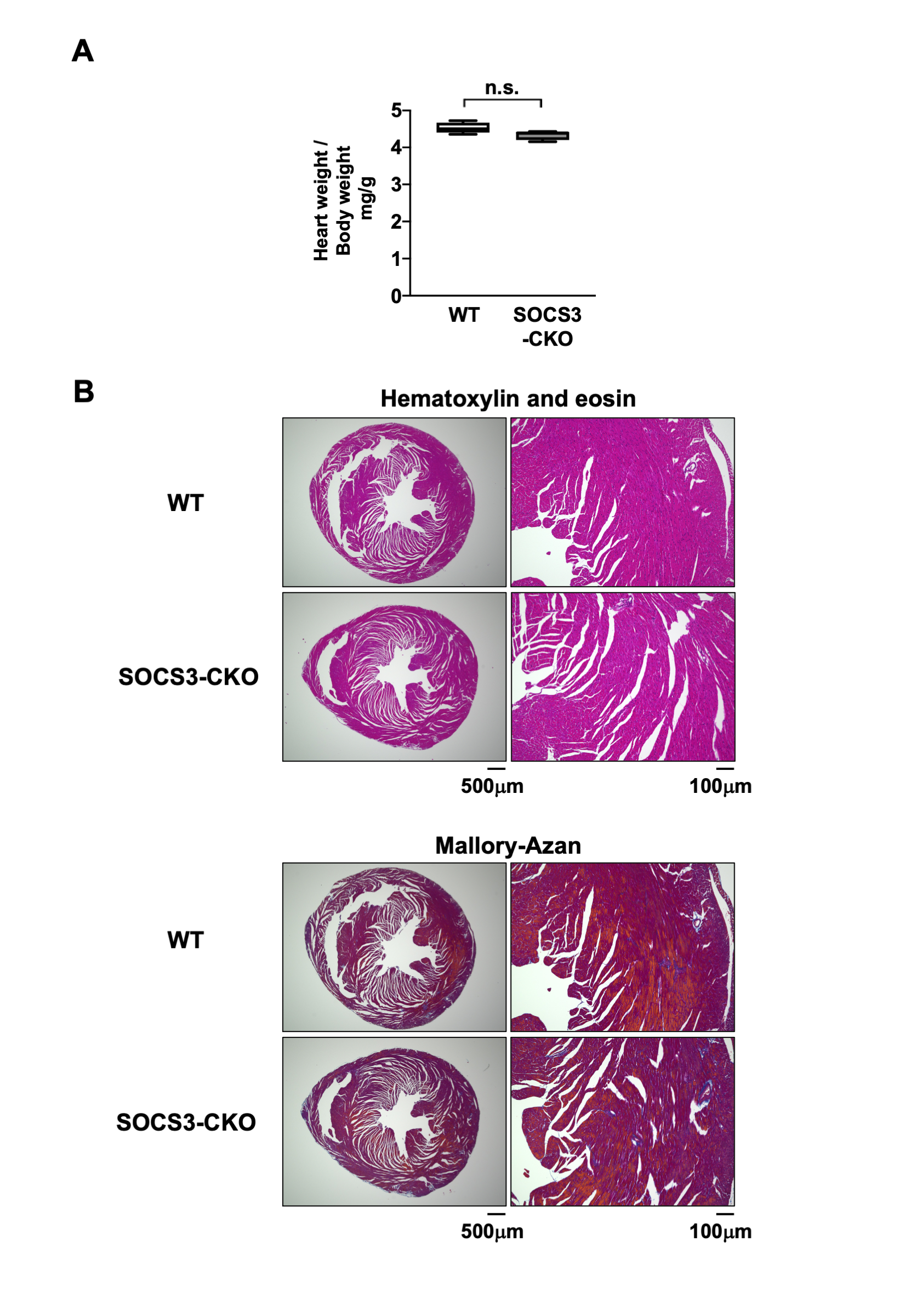

Supplement: S1 Fig — (A) Graph shows the ratio of heart weight-to-body weight based on intact hearts (n = 5 per group, Wilcoxon rank-sum test). (B) Sections from intact hearts of WT mice and SOCS3-CKO mice were stained with hematoxylin and eosin and Mallory-Azan (n = 5 per group). (TIF) [file pone.0254712.s001.tif]

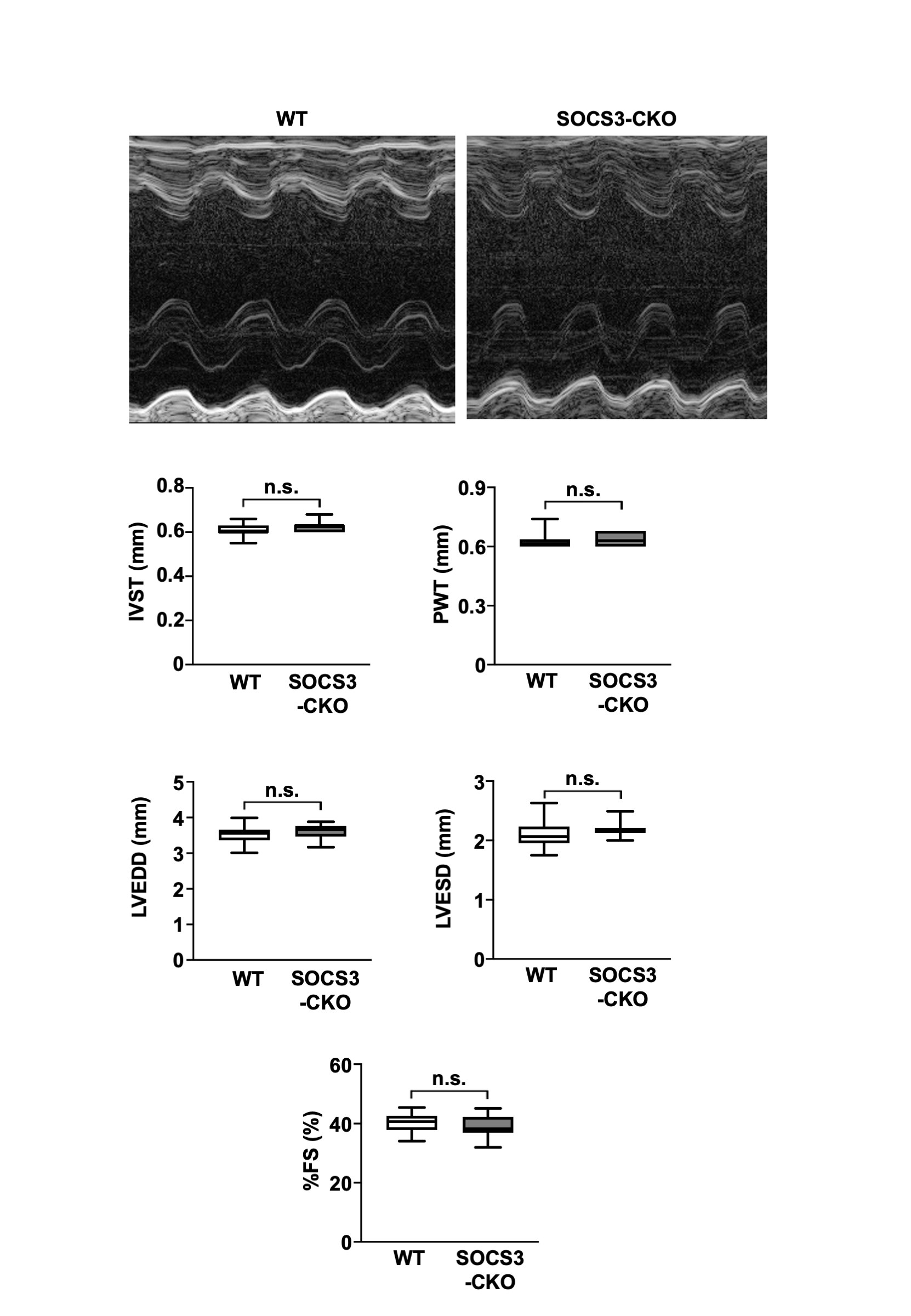

Supplement: S2 Fig — Echocardiography was performed in intact WT mice and SOCS3-cKO mice (n = 11–14 per group, Wilcoxon rank sum test). IVST, interventricular septum thickness; PWT, posterior left ventricular wall thickness; LVEDD, left ventricular end-diastolic diameter; LVESD, left ventricular end-systolic diameter; FS, fractional shortening. (TIF) [file pone.0254712.s002.tif]

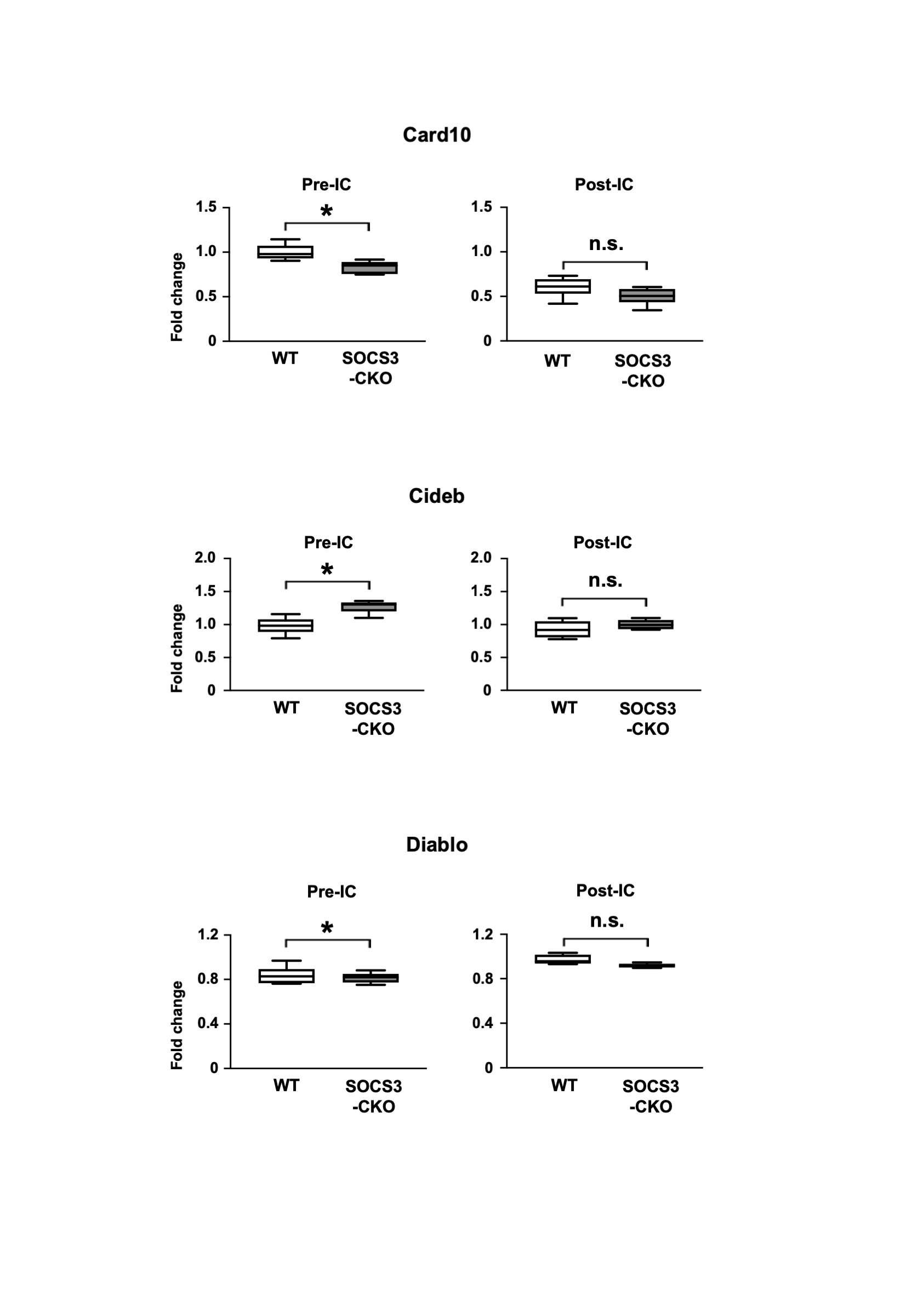

Supplement: S3 Fig — We prepared mRNA from heart tissue of WT or SOCS3-CKO mice, obtained pre-IC and 5 h after IC. This mRNA was subjected to real-time PCR analysis. Values are expressed as fold change relative to the values from WT mice of pre-IC (n = 5–6 for each group). *p < 0.05 (Wilcoxon rank-sum test). Card10 (Caspase recruitment domain family, member 10), Cideb (Cell death-inducing DNA fragmentation factor, alpha subunit-like effector B), Diablo (Diablo homolog, Drosophila). (TIF) [file pone.0254712.s003.tif]

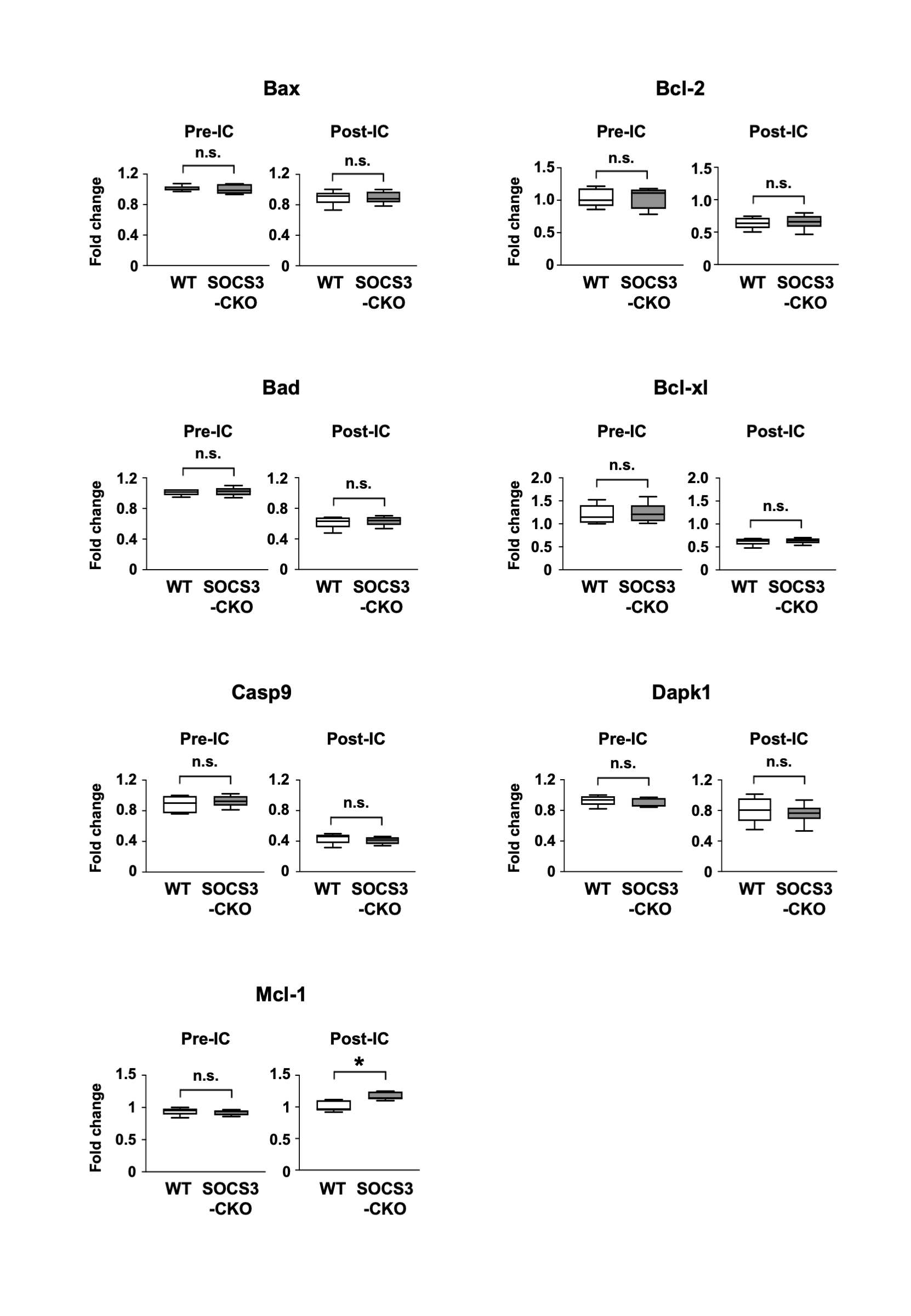

Supplement: S4 Fig — We prepared mRNA from heart tissue of WT or SOCS3-CKO mice, obtained pre-IC or 5 h after IC. This mRNA was subjected to real-time PCR analysis. Values are expressed as fold change relative to the values from WT mice of pre-IC (n = 5–6 for each group). *p < 0.05 (Wilcoxon rank-sum test). Bax (Bcl2-associated X protein), Bcl-2 (B-cell leukemia/lymphoma 2), Bad (BCL2-associated agonist of cell death), Bcl-xl (Bcl2-like 1), Casp9 (Caspase 9), Dapk1 (Death associated protein kinase 1), Mcl-1 (Myeloid cell leukemia sequence 1). (TIF) [file pone.0254712.s004.tif]

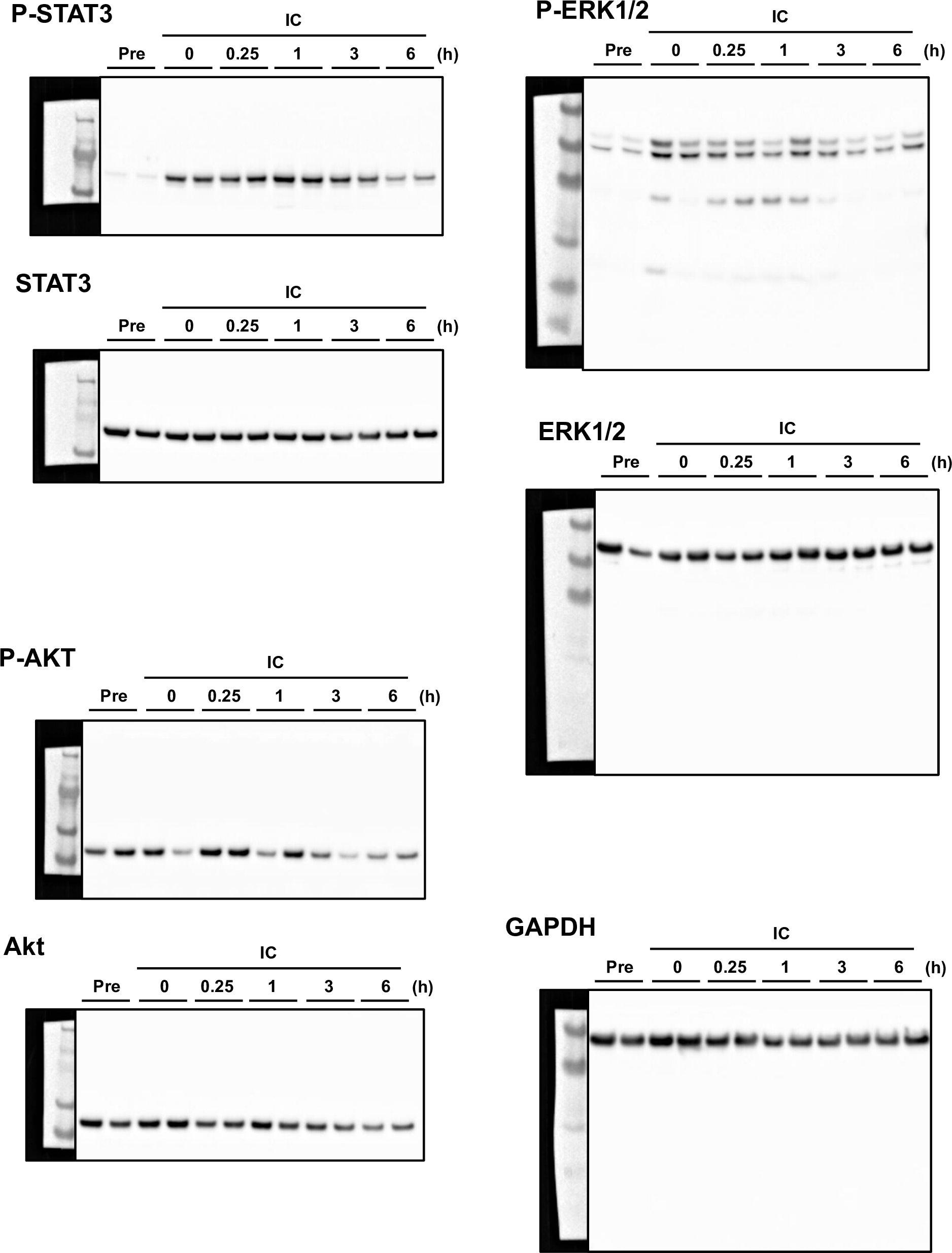

Supplement: S5 Fig — Blots were probed using antibodies against tyrosine- phosphorylated STAT3 (P-STAT3), STAT3, phosphorylated AKT (P-AKT), AKT, phosphorylated ERK (P-ERK1/2), and GAPDH. (TIF) [file pone.0254712.s005.tif]

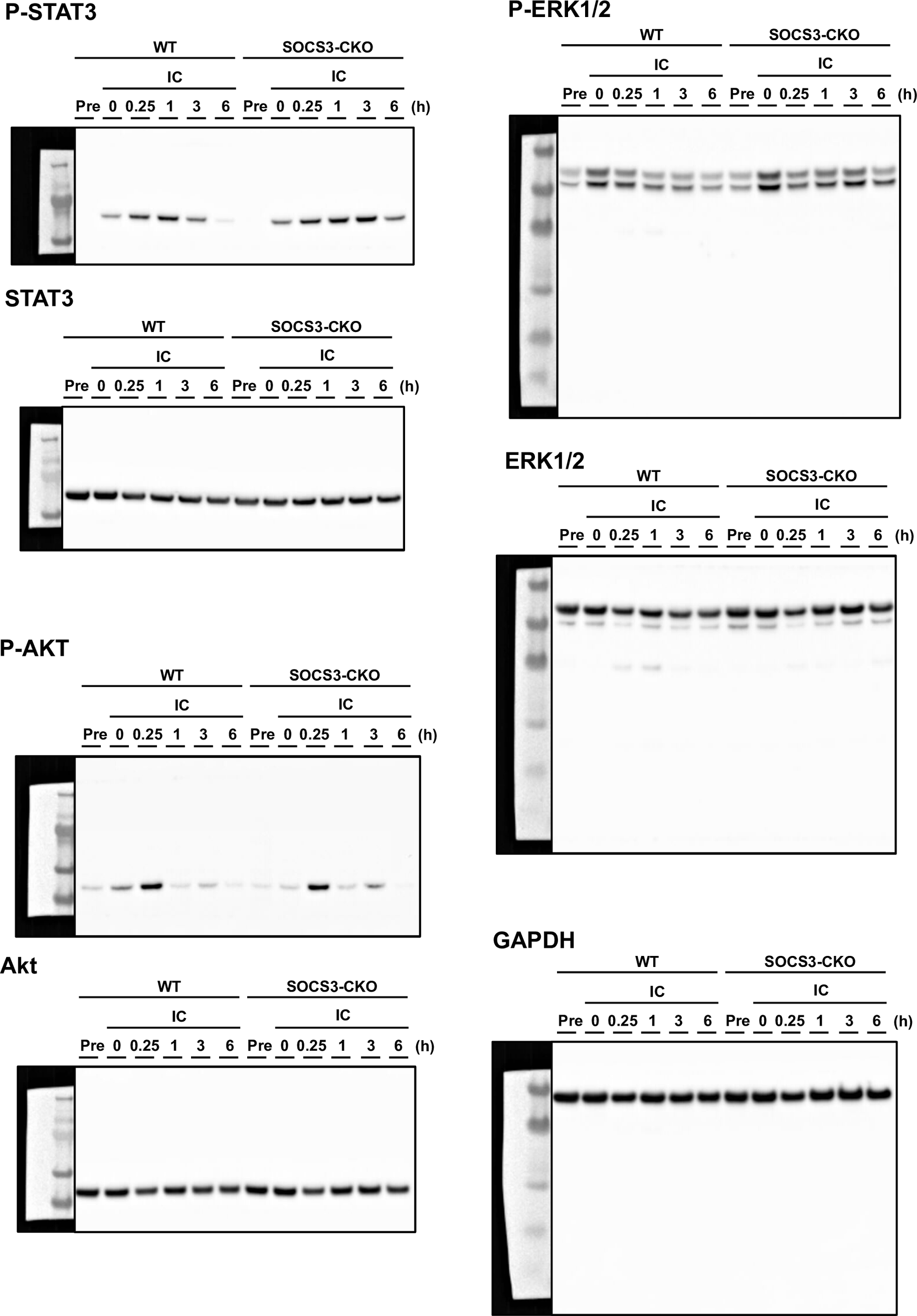

Supplement: S6 Fig — Blots were probed using antibodies against tyrosine- phosphorylated STAT3 (P-STAT3), STAT3, phosphorylated AKT (P-AKT), AKT, phosphorylated ERK (P-ERK1/2), and GAPDH. (TIF) [file pone.0254712.s006.tif]

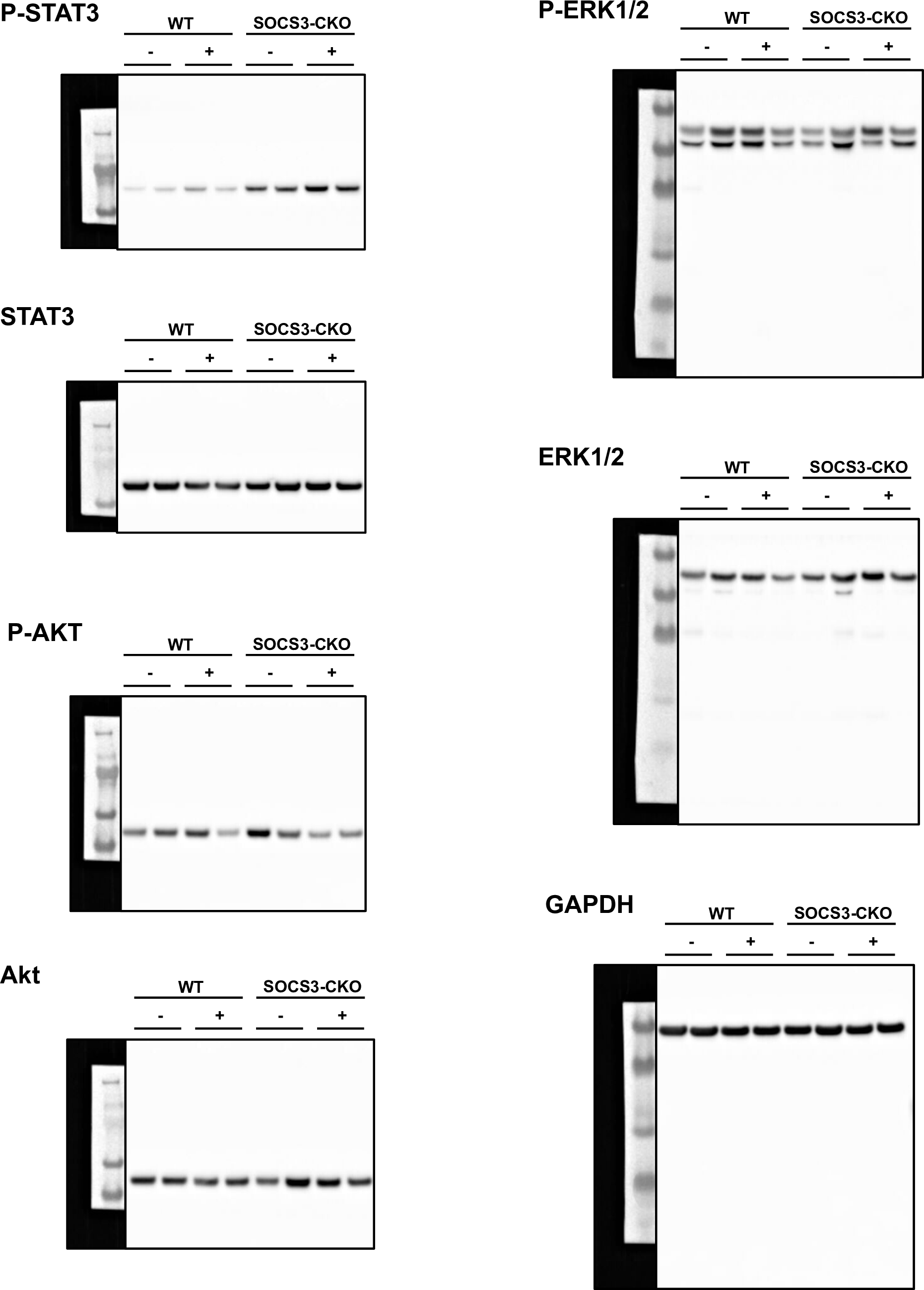

Supplement: S7 Fig — Blots were probed using antibodies against tyrosine- phosphorylated STAT3 (P-STAT3), STAT3, phosphorylated AKT (P-AKT), AKT, phosphorylated ERK (P-ERK1/2), and GAPDH. (TIF) [file pone.0254712.s007.tif]
